# Tumor Microenvironment–Responsive Polypeptide Nanogels for Controlled Antitumor Drug Delivery

**DOI:** 10.3389/fphar.2021.748102

**Published:** 2021-10-27

**Authors:** Yanhong Liu, Linjiao Chen, Qingyang Shi, Qing Zhao, Hongshuang Ma

**Affiliations:** ^1^ Center for Reproductive Medicine, Center for Prenatal Diagnosis, First Hospital, Jilin University, Changchun, China; ^2^ Department of Obstetrics, First Hospital, Jilin University, Changchun, China; ^3^ Department of Rheumatology and Immunology, The First Hospital of Jilin University, Changchun, China

**Keywords:** nanogels, stimulus-responsive, drug delivery, polypeptide, nanoparticle, copolymer

## Abstract

Tumor microenvironment–responsive polypeptide nanogels belong to a biomaterial with excellent biocompatibility, easily adjustable performance, biodegradability, and non-toxic properties. They are developed for selective delivery of antitumor drugs into target organs to promote tumor cell uptake, which has become an effective measure of tumor treatment. Endogenous (such as reduction, reactive oxygen species, pH, and enzyme) and exogenous (such as light and temperature) responsive nanogels can release drugs in response to tumor tissues or cells to improve drug distribution and reduce drug side effects. This article systematically introduces the research progress in tumor microenvironment–responsive polypeptide nanogels to deliver antitumor drugs and provides a reference for the development of antitumor nanoformulations.

## Introduction

Cancer is a kind of disease with the highest mortality rate in the world. Although a variety of cancer treatments have been developed, such as gene therapy (O'Connor and Crystal, 2006), photodynamic therapy (Chen et al., 2002), photothermal ablation (Lal et al., 2008), immunotherapy (Riddell, 2001), and radiation therapy (Stupp et al., 2005), chemotherapy combined with surgical treatment is one of the most effective ways to cure cancers. Chemotherapy to cure cancers mainly rises to the challenge to selectively eliminate tumor cells without affecting normal tissues. Ideally, an antitumor drug will target tumor cells and be kept within a range expected to achieve the therapeutic effect for a long time. To this end, researchers have conducted a lot of research in the field of nanomedicine (Wei et al., 2021; Zheng et al., 2021) and developed a series of nanocarrier materials, such as dendrimers (Liu et al., 2011), micelles (Zheng et al., 2020), nanogels (Ma et al., 2021), mesoporous silica (Zhang L. et al., 2016), and carbon dots (Zeng et al., 2016).

**FIGURE 1:**
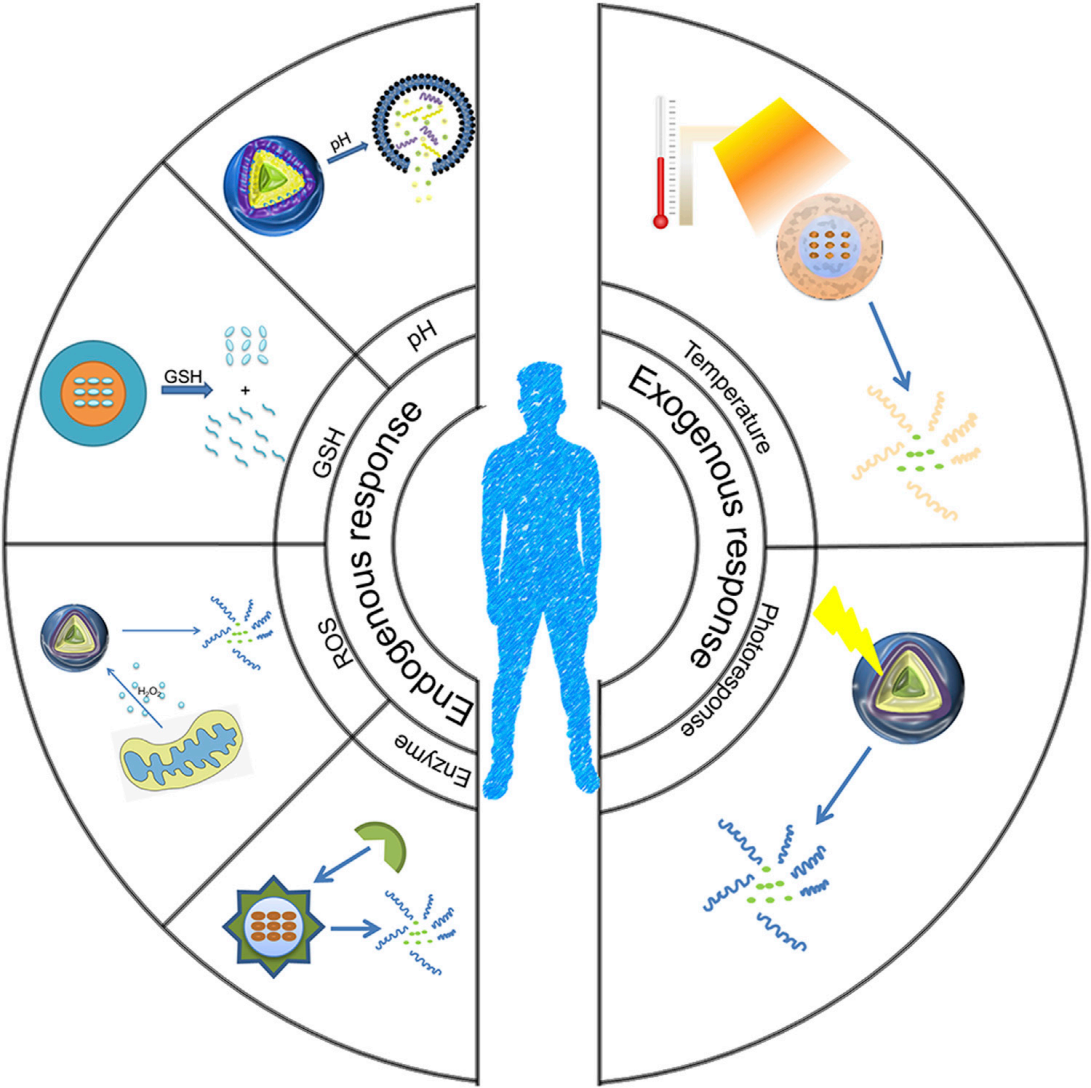
Schematic diagram of the environmentally responsive nanogels responding to environmental changes.

The ideal drug delivery system should maintain enhanced tumor accumulation, effective cell uptake, and a controlled drug release rate, thereby improving the efficacy of drug delivery and minimizing the side effects of drugs. Nanogels are nanoscale gel particles with a stable three-dimensional swelling polymer chain cross-linked network (Gonçalves et al., 2014; Merino et al., 2015), and the inclusion of drugs in them can effectively avoid enzymatic degradation. Nanogels have high hydrogel water content, can shrink or swell according to changes in the external environment, have good passive targeting, and have the characteristics of long blood circulation time. Due to the enhanced permeability and retention (EPR) effect, nanosized particles are more likely to accumulate in the interstices of tumors, and sizes less than 100 nm are ideal for cancer drug delivery (Ramasamy et al., 2014; Sundaramoorthy et al., 2016). In the tumor microenvironment, factors such as low pH, a high reactive oxygen species (ROS) level, high glutathione (GSH) concentration, and high enzyme expression in the environment will cause the linker to break and release the drug. As a result, a nanogel with the characteristics of pH, active oxygen, reduction sensitivity, enzyme, temperature, and light response was designed (Figure 1).

Compared with traditional polymer nanogels, peptide nanogels have excellent characteristics, such as good biocompatibility, easy adjustment of performance, and release of safe and non-toxic products after degradation. Compared with polyester nanogels (Bahadur and Xu, 2012; He et al., 2014), the acidity increases due to the accumulation of carboxyl-containing molecules after degradation. The degradation products of polypeptide nanogels are mainly amino acids, which will not cause further side effects (Ding et al., 2011a; Tian et al., 2012; Zhang et al., 2015). In addition, due to the rich variety of amino acids, a variety of monomers can be selected to adjust the properties of the polypeptide nanogels. At the same time, the side chain of the polypeptide can further modify other functional groups through reactions such as transesterification, condensation, click chemistry, or aminolysis to prepare polyamino acids with different reactivities and biological activities. Therefore, in all drug delivery systems, the use of environmentally responsive polypeptide nanogels as a promising method to deliver antitumor drugs has attracted widespread attention. In this article, the environmentally responsive nanogels are divided into three categories: endogenous response, exogenous response, and multiple responses, and elaborated on the research progress in this area that has been made in recent years (Table 1).

**TABLE 1:** Tumor microenvironment–responsive polypeptide nanogels for controlled antitumor drug delivery.

Material type	Material	Payload	Results	References
pH-resonsive	poly (l-glutamic acid)/chitosan (PLGA/CS)	mitoxantrone	The nanogel has very good biocompatibility, and the drug-loaded nanogel has a significant inhibitory effect on tumor cells compared with free drugs	Yan et al. (2017)
	mPEG-b-PLG	DOX	Compared with the free drug, it increases the apoptosis of tumor tissues, shows an enhanced therapeutic effect, reduces systemic toxicity, and also improves the biodistribution, pharmacokinetics, and efficacy of the drug	Li et al. (2013)
	poly (MEO2MA-OEGMA-mBISS)	DOX	The nanogel has good biocompatibility, and the cytotoxicity is similar to that of the free drug after loading the drug	Mackiewicz et al. (2019)
	PEG-PASP-DOX	DOX	It exhibits obvious phototoxicity and synergistic toxicity to tumor cells and accumulates for a long time in tumors	Lu et al. (2014)
	PGA	DOX	Optimal impact on primary tumor growth, lung metastasis, and toxicologic properties	Arroyo-Crespo et al. (2018)
	Methoxy poly (ethylene glycol)-b-poly [ N-[N-(2-aminoethyl)-2-aminoethyl] -l-glutamic acid]	DOX	The lethality of DOX in nanoparticles to cells is also higher than that of free DOX	Li et al. (2018b)
	MPEG-PEI-PBLL	DOX	Reduces systemic toxicity, has little effect on healthy cells, prolongs the circulation time, and improves the viability of mice	Yan et al. (2020)
Reduction-responsive	PEG_113_-b-PPAL	DOX	The nanogel has good biocompatibility and has a better inhibitory effect on tumor cells	Yao et al. (2019)
	STP-NG	SHK	Good safety and can enhance tumor-specific affinity	Li et al. (2018a)
	mPEG-P(LP-co-LC)	DOX	Shows enhanced antitumor efficacy and safety, limits the recruitment of regulatory T cells and myeloid suppressor cells, and enhances the antitumor activity of CD8 + T cells	Liu et al. (2015) He et al. (2016) Feng et al. (2021)
	PLL-P(LP-co-LC)	HCPT	Enhanced apoptosis	Guo et al. (2017)
	R_9_-PEG-P(LP-co-LC)	HCPT	Extend retention time and enhance drug penetration	Guo et al. (2020)
	PMNG	DOX	Good targeting and great advantages in inhibiting the growth of primary and metastatic tumors	Chen et al. (2017b)
	NIRF nanogel	DOX	Good accumulation in tumor cells	Xing et al. (2012)
	PEG-PCys-Pphe	DOX	Reduce the loss of drugs and quickly release drugs in response to intracellular GSH levels	Wang et al. (2012)
	DCM	DOX	Reduce drug efflux, significantly reduce tumor volume, and cause no damage to major organs	Deepagan et al. (2016)
ROS-responsive	PEG-b-PPGA	DOX	The structure is stable, the antitumor activity is better than that of free drugs, and the treatment tolerance is good	Kim et al. (2013)
Enzyme-responsive	mPEG-Peptide-PCL	DOX	Good targeting	Guo et al. (2018)
	Hep-F127	hydrated cisplatin and curcumins	High drug-loading efficiency and good slow and controlled release effect	Nguyen et al. (2018)
Temperature-responsive	PEG-PK-PA/HA	FITC-BSA	The nanogel can be tightened by heat shrinkage and high internalization efficiency	Ko et al. (2015)
	poly (N-isopropylacrylamide), poly (l-lactic acid), and poly (l-lysine)	NGF	It has no cytotoxicity to neuron-like PC12 cells for at least 1 month	Kim et al. (2018)
Light-responsive	SiPING	DOX	It has high delivery efficiency to prevent cell efflux and can release large amounts of drugs through near-infrared light	Chen et al. (2019)
	poly (Cys–PCL)	paclitaxel	It has excellent tumor suppression ability, good drug tolerance, and biological safety	Zhang et al. (2019)
	mPEG-b-P(LGA-co -CELG-dTbDEA)	DOX	It has good delivery effect, improved antitumor activity, and safety	Ding et al. (2013)
	Methoxy poly (ethylene glycol)-poly (l-glutamic acid-co-l-cystine)	DOX	It releases fast and shows excellent tumor suppression and safety	Shi et al. (2017)
	FD-NP	DOX	It can significantly improve cell binding and terminal/lysosome escape and high delivery efficiency *in vivo*	Yi et al. (2016)
	sPEG/GLC	miR155	Immunosuppressive TAM effectively repolarizes into antitumor M1 macrophages, increasing the activated T lymphocytes and NK cells in the tumor	Liu et al. (2017b)
	PC-g-PEG-LAC	DOX and 6-mercaptopurine	It has a targeting effect on the HepG2 cell line and has enhanced cytotoxicity compared to a single free drug	Wu et al. (2016)
	SNP	CA4P and BTZ	It reversed the drug resistance caused by the overexpression of ABCG2 and significantly inhibited tumor growth	Chen et al. (2020)

## Nanogel Synthesis Method

The preparation method of the polyamino acid nanogel is relatively simple and is usually divided into three kinds: 1) chemical cross-linking, 2) monomer polymerization, and 3) template method.

Chemical cross-linking refers to the formation of a cross-linked structure by connecting a polypeptide containing necessary side groups to a molecule with a multifunctional group and finally forming a nanogel. This is one of the most extensive methods used for preparing peptide nanogels. The side chain of the polypeptide can be further modified with other functional groups through reactions such as transesterification, condensation, click chemistry, or aminolysis to prepare polyamino acids with different reactivities and biological activities.

Monomer polymerization refers to the one-step ring opening polymerization (ROP) of amino acid monomers with two N-internal carboxylic anhydride groups (NCA) through a one-step polymerization method to obtain polyamino acid nanogels. There are not many amino acids that can be used in this method, and they are mainly used in reduction-responsive polypeptide nanogels.

The template method is used to construct a template so that the nanogel can form a shell on the surface and then eliminate the template to obtain a polypeptide nanogel. This method uses fewer, commonly used templates including gold nanoparticles, mesoporous silica, and so on.

These three methods have opened a broad spectrum for the synthesis of peptide nanogels and made the usage of responsive peptide nanogels possible.

## Single Stimulus–Responsive Nanogels

### Endogenous Tumor Microenvironment–Responsive Nanogels

Endogenous tumor microenvironment–responsive nanogels are a group of nanogels that selectively respond to the difference between the internal tumor environment and normal tissues and then release the loaded drug to avoid poor water solubility, poor distribution, and high toxicity of chemotherapeutic drugs to normal tissues. The common ones are mainly classified as follows:

#### pH-Responsive Nanogels

The upregulation of glycolysis, a near-universal trait of primary and metastatic cancers indicated based on clinical tumor imaging can give rise to glucose consumption and the production of metabolite lactic acid, which in turn leads to microenvironmental acidosis. Significantly different pH values between plasma (pH = 7.4) and the tumor extracellular microenvironment (pH = 6.5–7.2) and between lysosomes (pH = 4.5–5.5) and endosomes (pH = 5.5–6.8) (Kuppusamy et al., 2002; Wojtkowiak et al., 2011) provide a good trigger for drug release. From this observation, a pH-responsive drug delivery system has been developed and deeply explored. The existing preparation strategies mainly include hydrophilic–hydrophobic conversion and the introduction of pH-sensitive bonds.

The tumor extracellular microenvironment is weakly acidic due to the combination of acidic byproducts produced via tumor metabolism and high glycolytic activity due to impaired acid clearance, where the pH-responsive groups are protonated to trigger hydrophilic–hydrophobic conversion, separating drug molecules from the carrier and releasing them in the tumor tissue, thereby improving the efficiency of nanoparticle delivery.

**FIGURE 2:**
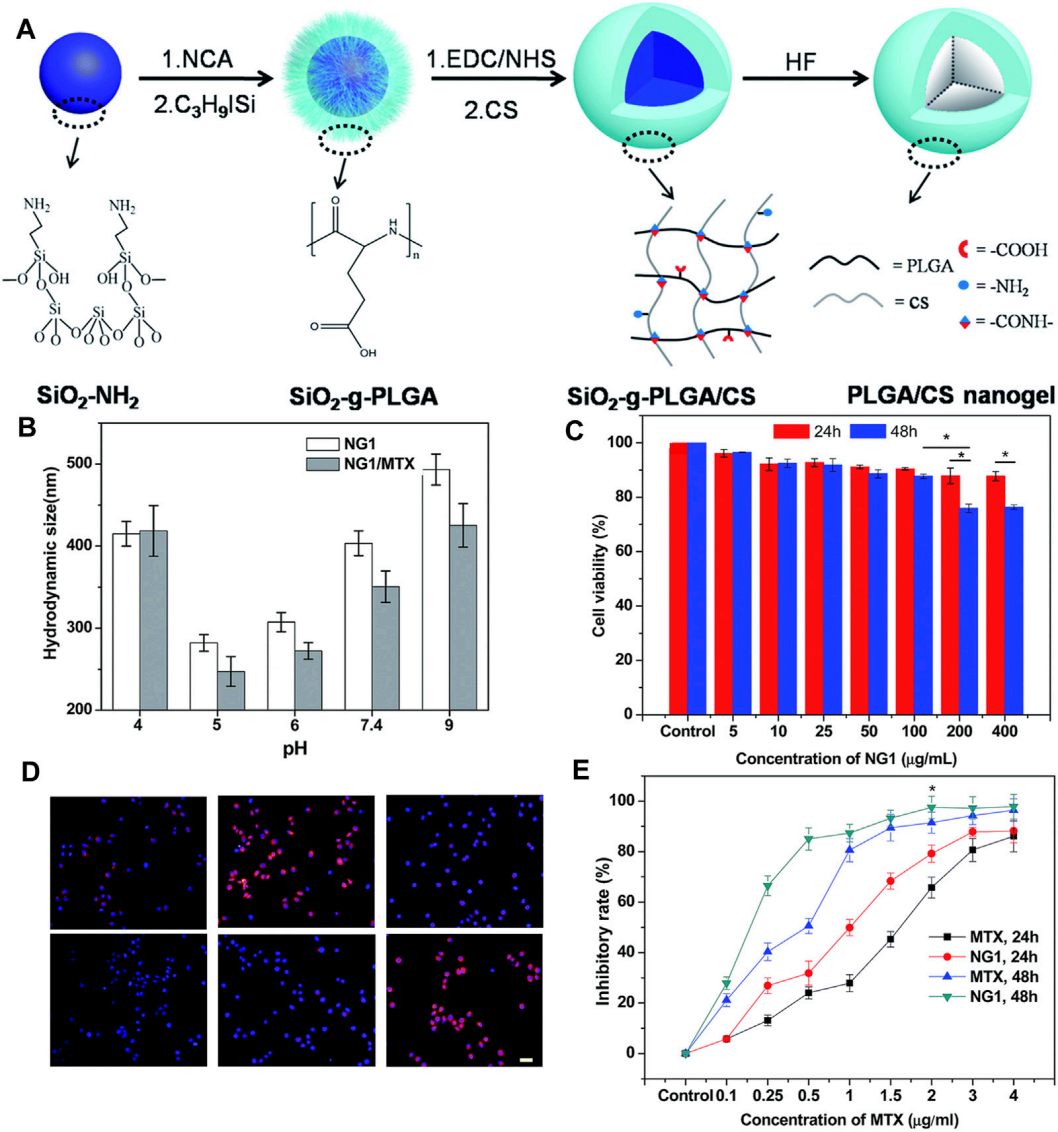
Physical properties of PLGA/CS and the action of drugs (Yan et al., 2017). **(A)** Synthesis method of nanogels. **(B)** Change in particle size of nanogels at different pH. **(C)** Endocytosis effect of nanogels. **(D)** Inhibition rate of MTX-loaded nanogel and MTX on tumor cells.

Yan et al. (Yan et al., 2017) reported a method to synthesize a hollow nanogel poly (l-glutamic acid)/chitosan (PLGA/CS) loaded with the water-soluble antitumor drug mitoxantrone using a nanotemplate method with good biocompatibility. As shown in Figure 2A, the author grafted PLGA onto silica nanoparticles and then used CS as a cross-linking agent to finally remove the template silica. As shown in Figure 2B, the nanogel has a particle size of 189.3 nm under neutral conditions, and the size of the nanogel is significantly reduced when the pH value is 5–9. As shown in Figure 2C, the use of the MTT assay to study the cytotoxicity of PLGA/CS nanogels and drug-loaded nanogels *in vitro* shows that the biocompatibility of nanogels is very good, and the MTX-loaded nanogels perform well on tumor cells compared with free MTX, which shows a significant inhibitory effect. As shown in Figure 2D, the endocytosis of nanogels was evaluated using a fluorescence microscope. Rhodamine B (RB) was used as a model dye. The fluorescence signal of cells incubated with RB-loaded nanogels was much stronger than that of RB-treated cells. This is because CS decorated on the surface of the nanogel can promote cell internalization. This will deliver higher concentrations of drugs into the cells (Wang et al., 2016). Therefore, this pH-sensitive nanogel synthesized by the nanotemplate method has great potential in smart drug delivery systems (Weigel and Yik, 2002).

Li et al. (Li et al., 2013) developed a polypeptide-based block ionomer complex composed of anionic methoxy( polyethylene glycol)-b-poly-l-glutamic acid and cationic doxorubicin hydrochloride for treating non–small cell lung cancer.

Compared with normal biological tissues, solid tumors can cause cell acidosis. Therefore, it is a current hotspot for introducing pH-sensitive bonds into nanogels.

Mackiewicz et al. (Mackiewicz et al., 2019) synthesized biocompatible degradable nanogels based on polyethylene glycol methyl methacrylate (OEGMA) and diethylene glycol methyl methacrylate cross-linked with redox-sensitive linker N,N-bis (methacryloyl) cystine to synthesize biocompatible and degradable nanogels. With the aid of the carboxyl group in N,N-bis (methacryloyl) cystine, the nanogels had become sensitive to pH, became more stable in the physiological environment, and binded 8% DOX loaded through electrostatic interaction. This degradable nanogel was non-toxic to cells; however, the drug-loaded nanogel has a killing effect on cancer cells, similar to free DOX.

Lu et al. (Lu et al., 2014) formed a pH-responsive peptide nanogel by the hydrazone self–cross-linking of poly (asparagine sultaine-co-aspartyl hydrazide) and poly (asparagine sultaine-co-2-oxyethyl aspartame) under mild conditions. Hydrazone has been extensively studied in biomedical research due to its preparation under mild conditions and hydrolysis under acidic conditions (Bae and Kataoka, 2009). This nanogel has excellent stability and anti-protein adsorption ability. The nanogel has been stable in the PBS solution for 3 weeks and in the protein solution for at least 12 h. When the pH drops to 4.0, the size of the PAsp nanogel will change rapidly. With 18% drug loading rate, doxorubicin (DOX) as a model drug has been loaded onto the nanogel, and the potential of the nanogel as a pH-sensitive drug delivery system was evaluated. When pH = 5.0, the nanogel released more than 70% of the drug in 12 h, and only about 25% of the drug was released at a pH of 7.4. In the *in vitro* cytotoxicity test, the nanogel showed good biocompatibility. Confocal microscopy results show that the loaded DOX can be successfully released and transported to the nucleus of the tumor cells. Therefore, this easily prepared nanogel is a promising, intelligent drug delivery system.

Arroyo-Crespo et al. (Arroyo-Crespo et al., 2018) developed a family of biodegradable poly (l-glutamic acid) (PGA) conjugates based on pH response, to optimize anticancer effects. DOX-loaded conjugates prepared using different pH-sensitive linkers to connect DOX and amino glutamine could promote specific drug release from the main polymer chain in the tumor microenvironment (TME). In a preclinical metastatic triple-negative breast cancer (TNBC) mouse model, low DOX load and short linkers have the best effects on primary tumor growth, lung metastasis, and toxicologic properties. Juan et al. (Arroyo-Crespo et al., 2018) also determined the relevant molecular mechanisms through transcriptomics analysis, including differential immunomodulation between the conjugates and cell death pathways. Their efforts focus on the advantages of the targeted TME, the therapeutic value of polymer-based combined methods, and methods based on omics analysis that can enhance the anticancer performance of drug delivery materials.

**FIGURE 3:**
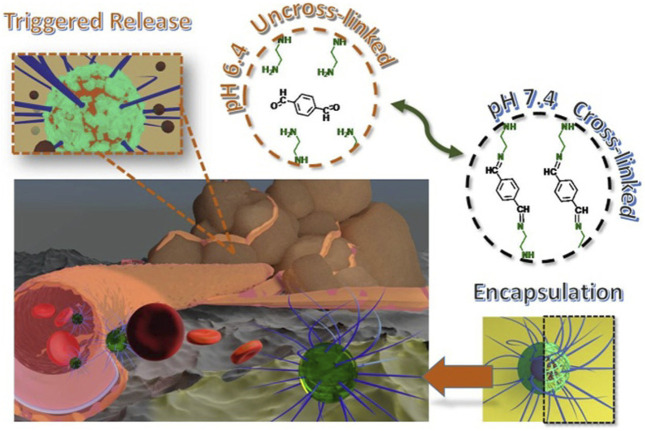
Formation of pH-responsive mPEG-b-PNLG nanogels and the mechanism underlying drug delivery (Li Y. et al., 2018).

Li et al. (Li Y. et al., 2018) proposed a new method to prepare pH-responsive nanogels by a simple one-step method. They used hydrophilic methoxy poly (ethylene glycol)-b-poly {N-[N-(2-aminoethyl)-2-aminoethyl]-l-glutamic acid} to construct a pH-responsive nanogel by a pH-sensitive benzimine bond. The acid-labile benzimine bond could be cleaved under weak acidic conditions (pH = 6.4) (Figure 3), and then the prepared nanogel could physically encapsulate the therapeutic molecules with high stability with the help of the mPEG shell, whereas, the core of the nanogel at physiological pH could minimize the non-specific absorption of its payloads and side effects. Once the drug-loaded nanogel system accumulated at the tumor site was exposed to the acidic conditions of the tumor, the core part could be quickly destroyed because of the rupture of the benzimine bond, resulting in enhanced drug accumulation at the tumor site. Compared with other pH-responsive nanogels, this nanogel has advantages in three aspects: 1) Preparation and drug loading of the nanogel can be completed in one step; 2) the polymer used is biodegradable and biocompatible, and the nanogel responds to subtle pH changes; and 3) a decrease in pH will trigger the release of the loaded drug and the dissociation of the nanogel, which makes it possible to remove the nanogel that has completed its mission.

Yan et al. (Yan et al., 2020) successfully synthesized a pH-responsive nanomaterial methoxy poly (ethylene glycol)-b-polyethyleneimine-poly (N_ε_-Cbz-l-lysine) (mPEG-PEI-PBLL), further improving its controlled release ability and drug loading efficiency. Schiff’s base introduced into the polymer allowed the material to be responsive to changes in pH. mPEG-PEI-PBLL was degraded at pH = 5.0 and stabilized at pH = 7.4. mPEG-PEI-PBLL could self-assemble due to its amphiphilic property and effectively encapsulates the therapeutic agent by introducing PEI (hyperbranched copolymer). The drug loading index of the nanogel was about 25.8%. The PBLL block as a hydrophobic block can obtain a good degree of polymerization, and the polymer structure which was similar to the protein has very good biocompatibility Moreover, therapeutic agents such as DOX and DiD, a near-infrared dye, were effectively contained in the pH-responsive nanogels. In this study, the biological properties of mPEG-PEI-PBLL nanogels were also evaluated by investigating *in vivo* cell absorption, biocompatibility, and pharmacokinetics. Cell uptake studies indicated that the absorption rate of DOX which was wrapped in the nanogels was much slower than free DOX, subsequently reducing systemic toxicity in the body. The biodistribution of the drug shows that nanogels can reduce excessive damage to healthy cells and tissues. Moreover, *in vivo* treatment and pharmacokinetic studies indicated that the nanogels could accumulate at the tumor tissue to inhibit the growth of the tumor while improving the survival of mice. These findings reveal that the drug delivery system prolongs the circulation of the drug in the blood and allows the drug to effectively accumulate at the tumor tissue.

Therefore, the nanogels prepared by these two strategies have very broad application prospects.

#### Reduction-Responsive Nanogels

GSH is an important chemical substance to determine the redox environment in the body. The concentration of GSH in normal tissue cells (2–10 mmol/L) is 100–1,000 times that of the extracellular concentration. Because tumor cells are often deprived of oxygen, the concentration of GSH in tumor cells is at least four times higher than that in normal tissues and cells. Therefore, the tumor cell microenvironment has strong reducibility (Meng et al., 2009; Park et al., 2010; Sun et al., 2013; Huo et al., 2014; Phillips and Gibson, 2014) To this end, numerous studies have been conducted.

Cross-linking is the main component to prepare reduction-responsive nanogels. These kinds of agents are active units that contain disulfide bonds that can be reduced and form a 3D cross-linked redox-responsive network through the disulfide bond to load therapeutic drugs. These nanogels can be reduced by the reducing environment, thereby releasing their payload followed by biodegradation. Many redox reactive crosslinkers used in the synthesis of nanogels contain cystine and cystamine as reductive reactive components, terminal acrylates are involved in free radical polymerization reactions, and PEG was used as the functional backbone of the nanogels (Ma et al., 2010; Yu et al., 2011; Dispinar et al., 2012; Zhang Q. et al., 2013). Disulfide bonds are the main reporting structural units and important structural unit of reduction-reactive materials, which have excellent activity. When GSH or dithiothreitol are present, disulfide bonds will be broken to provide biodegradability and rapid drug release. To this end, disulfide bonds have been used to develop many reduction-responsive nanogels (Wu et al., 2009; Abdullah Al et al., 2013). This section mainly focuses on disulfide-bonded cross-linked nanogels from the perspective of cross-linking agents.

The first strategy is to prepare a disulfide bond cross-linked polypeptide nanogel by the addition of a disulfide bond–containing cross-linking agent into the polypeptide.

**FIGURE 4:**
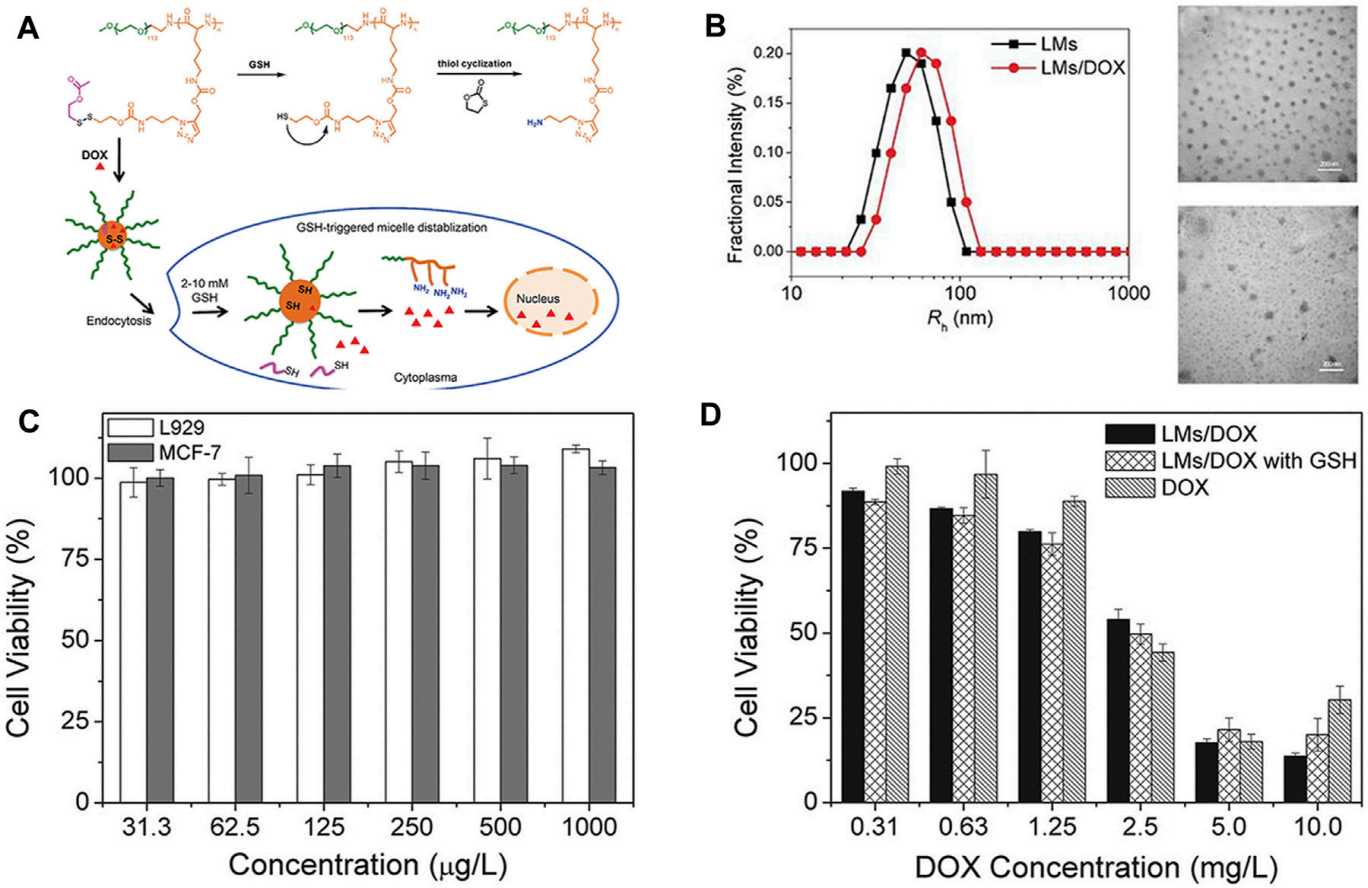
Chemical structure of the PEG_113_-bP (Lys-DSA) polymer thiol reaction and *in vitro* and *in vivo* experimental characterizations (Yao et al., 2019). **(A)** Schematic diagram of the chemical structure of the polymer thiol reaction and the reduction-sensitive DOX release behavior after LMs/DOX endocytosis. **(B)** Hydrodynamic radius and TEM images of blank and drug-loaded nanogels. **(C)** Cytotoxicity of nanogels on L929 cells and MCF-7 cells *in vitro*. **(D)** Cytotoxicity of DOX-loaded nanogels and free DOX.

Yao et al. (Yao et al., 2019) developed a polypeptide nanogel, poly (ethylene glycol)-block-poly (ϵ-propargyloxycarbonyl-l-lysine) (PEG_113_-b-PPAL) coupled with reducible side chains to deliver antitumor drugs. PEG_113_-b-PPAL was synthesized by ROP of alkyne-containing NCA. Then, they introduced the disulfide bond–containing side chain into the PEGylated polypeptide by the click reaction. The obtained polymer self-assembled to form a nanogel and exhibited a reduction reaction when treated with 10 mmol/L GSH (Figure 4A). Then, DOX was loaded onto the nanogel at a percentage of 6.73wt and a loading efficiency of 40.3%. The average diameters of the blank and polylysine-derived polymer nanogel loaded with DOX (LMs/DOX) were about 48.0 and 63.8 nm, respectively (Figure 4B). *In vitro* drug release shows that doxorubicin can be released much quickly in the presence of GSH. CLSM and flow cytometry analysis further proved the sensitive release behavior of LMs/DOX to GSH and its ability to enhance the intracellular delivery of DOX. MTT results showed negligible cytotoxicity of the polymer to normal cells L929 or cancer cells MCF-7 (Figure 4C). With the increase of DOX concentration, there was a better anti-proliferative activity in MCF-78 cells in LMs/DOX, LMs/DOX/GSH, and DOX HCl groups. When the concentration of DOX was lower than 2.5 mg/L, the cell viability of the nanogel group was lower than the free-drug group, and the cell viability of LMs/DOX with GSH was lower than LMs/DOX. This may be due to the fact that GSH pretreatment triggers faster release of DOX, resulting in a stronger inhibition of proliferation in MCF-7 cells (Figure 4D). These findings indicate that LMs/DOX have broad prospects in cancer treatment.

The second strategy is to use cystine with a disulfide bond to polymerize and to prepare cross-linked polypeptide nanogels.

**FIGURE 5:**
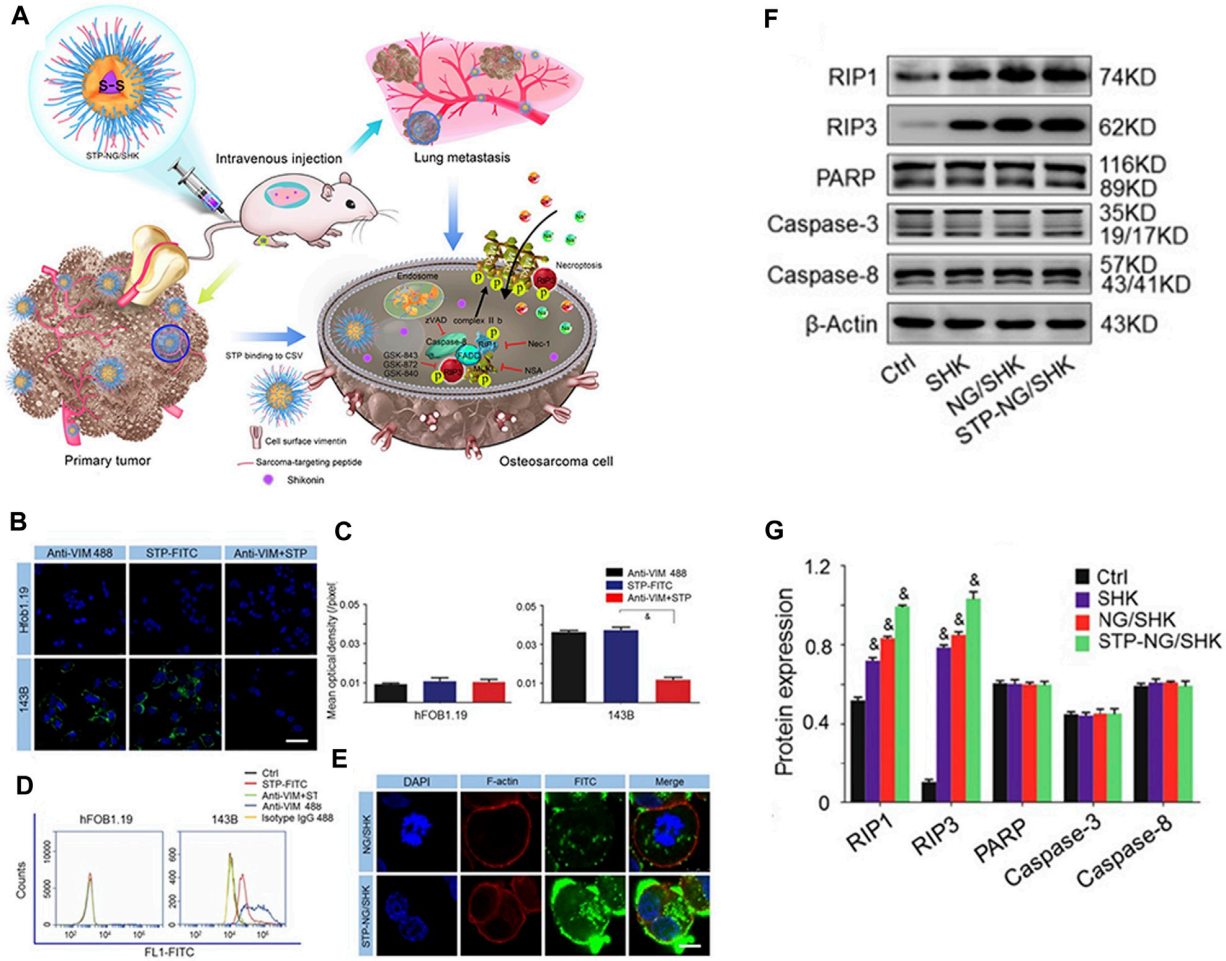
Schematic diagram of the nanogel system and cell experiments (Li S. et al., 2018). **(A)** Schematic diagram of the role of STP-NG/SHK in tumor and lung metastasis. **(B)** CLSM cell surface staining analysis of cell surface vimentin. **(C)** Semi-quantitative analysis of Figure B. **(D)** Immunologic evaluation of STP binding to cells with flow cytometry. **(E)** Cell uptake of NG/SHK-FITC and STP-NG/SHK-FITC. Western blot **(F)** and semi-quantitative analysis **(G)** of the cells.

**FIGURE 6:**
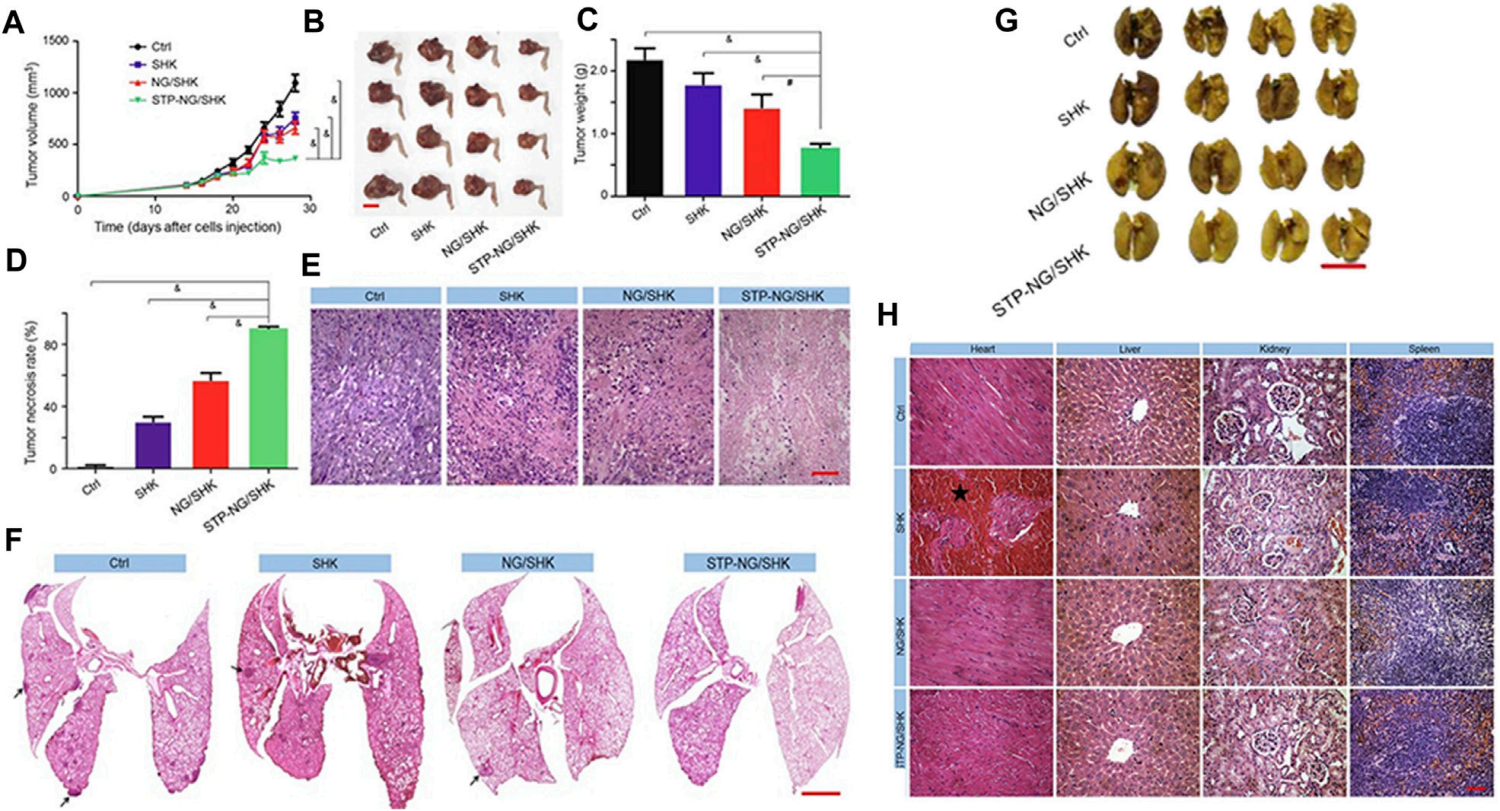
Animal experiments regarding tumor inhibition by the nanogels (Li S. et al., 2018). **(A)** Tumor growth curve. **(B)** Hind limb tumors. **(C)** Average weight of the osteosarcoma. **(D)** Calculation of tumor necrosis size. **(E)** Hematoxylin–eosin staining of the primary tumor. **(F)** H&E staining of the lung metastases. **(G)** Appearance of the lungs in each group. **(H)** H&E staining of mouse organs.

Li et al. (Li S. et al., 2018) attempted to reinforce the inhibition of osteosarcoma and lung metastasis by drug-induced necrosis. As shown in Figure 5A, they used a sarcoma-targeting peptide-modified disulfide-bonded cross-linked polypeptide nanogels (STP-NG) to enhance the intracellular delivery of shikonin (SHK), thereby inhibiting the progression of osteosarcoma with minimal systemic toxicity. This study confirmed the ability of STP to target 143B cells. As shown in Figures 5B–D, STP could specifically bind to cell surface vimentin in osteosarcoma, but does not bind to cell surface vimentin–deficient hFOB1.19 cells and blood cells (Satelli et al., 2014). Due to this difference, an ideal efficacy and minimal toxicity can be obtained. In addition, due to the targeting effect of STP-NG/SHK, higher membrane binding force has been observed, thereby increasing the cellular uptake of NG/SHK that could selectively accumulate in 143B osteosarcoma *in situ* (Figure 5E). RIP1- and RIP3-dependent necrosis *in vitro* proved that the inhibitory effect of STP-NG/SHK on cell proliferation was enhanced (Figures 5F,G). This animal experiment showed excellent antitumor efficacy of STP-NG/SHK (Figures 6A–E), and metastatic osteosarcoma in the STP-NG/SHK group was mostly suppressed (Figures 6F,G). These data prove that STP-NG/SHK profoundly inhibits osteosarcoma metastasis to the lungs. This excellent anti-metastatic effect was caused by intracellular drug release and tumor targeting. STP-NG/SHK targeting cell surface vimentin was related to epithelial-to-mesenchymal transition, and it is critical for metastasis (Satelli and Li, 2011). As shown in Figure 6H, SHK mainly damages the myocardium, whereas the abnormalities in other organs were minimal. H and E staining showed that compared with free SHK, NG/SHK and STP-NG/SHK showed much lower myocardial damage. These results indicate that coating drugs with nanogels can reduce systemic toxicity and improve biological safety, and STP can further effectively enhance tumor-specific affinity. Therefore, the intelligent drug delivery system equipped with SHK has great significance for VIM-targeted malignant tumor chemotherapy.

Feng et al. (Feng et al., 2021) synthesized three kinds of mPEG-P (LP-co-LC) nanogels with different l-cysteine scales, including mPEG-P (LP_10_-co -LC_15_) (NG_10-15_), mPEG-P (LP_10_-co-LC_10_) (NG_10-10_), and mPEG-P (LP_10_-co-LC_5_) (NG_10-5_). They evaluated the pharmacokinetic properties, tissue distribution, and antitumor efficiency of these nanogels with similar surface charge, morphology, and reductive reaction characteristics. As the degree of polymerization of l-cystine in the nanogels increased from five to 15, the size of the nanogels also increased from 105 to 256 nm. Researchers found that NG_10-5_ has the best stability in physiological environments. Compared with other two nanogels, NG_10-5_ could migrate to the tumor faster and accumulate for a longer time after intravenous injection. These results indicate that the size of the nanoparticles was more influential than that of polymers. Compared with the other two nanogels, NG_10-5_/DOX maintained a higher blood concentration and retained more drugs in the tumor. The improvement in the pharmacokinetics of NG_10-5_/DOX may result from slower liver clearance and more accumulation in the tumor. In addition, among these three preparations, NG_10-5_/DOX has optimal antitumor efficacy on 4T1 breast cancer. On this basis, Feng et al. (Feng et al., 2021) used mPEG-P (LP-co-LC) to co-encapsulate DOX and 1-methyl-DL-tryptophan (1 MT) referred as NG/(DOX + 1 MT) for 4T1 breast cancer treatment. By reducing the dose of DOX required to induce immunogenic cell death–inducing (ICD), the author achieved the goal of reducing the toxicity of mice and inducing the ICD of cancer cells at the same time. The drugs in NG/(DOX + 1 MT) were released in the tumor cells at the same time, and they have a synergistic antitumor effect. Nanogels exhibit excellent performance by downregulating the expression and activity of indoleamine 2,3-dioxygenase, limiting the recruitment of regulatory T cells and myeloid suppressor cells, and enhancing the antitumor activity of CD8^+^ T cells (Holmgaard et al., 2015; Peng et al., 2018; Huang et al., 2019).

Liu et al. (Liu et al., 2015) prepared a reduction-reactive nanogel methoxy poly (ethylene glycol)-poly (l-phenylalanine-co-l-cystine) (mPEG-P (LP-co-LC)) with a medium drug loading of 10.2 wt% and a diameter of less than 110 nm. DOX was used as a model antitumor drug loaded onto the nanogels to treat liver cancer in rodent models. The drug-loaded nanogels accumulated in the tumor tissues and released their payload rapidly due to the redox environment in tumor cells after the nanogels entered the cells. The toxicity of the loaded drug was mitigated because of stable encapsulation with the nanogels, and less drug leakage enhanced the retention and permeability effect. Compared with free drugs, the selective intratumoral release of DOX targeting intracellular reduction enhanced the antitumor efficacy of the drug-loaded nanogels.

He et al. (He et al., 2016) used mPEG-P (LP-co-LC) nanogels for RM-1 prostate cancer chemotherapy. DOX acted as a conventional chemotherapeutic drug to be embedded in the nanogels. Based on the drug-loaded nanogels labeled as NG/DOX, GSH induced cell swelling and promoted the release of DOX. Subsequently, NG/DOX presented with effective cell uptake and proliferative inhibition. In addition, NG/DOX showed enhanced antitumor efficacy and safety in the mouse model of RM-1 prostate cancer transplantation, indicating that it has a huge therapeutic potential in clinical practice.

In the study by Guo et al. (Guo et al., 2017), poly (l-lysine)-poly (l-phenylalanine-co-l-cystine), delivered 10-hydroxycamptothecin (HCPT) into BC cells *in situ*. The drug-loaded nanogel was labeled as NG/HCPT. NG/HCPT increases retention time and improves tissue permeability, thereby promoting cancer cell uptake and intracellular activation of HCPT. Positively charged poly (l-lysine) allows the nanogel to bind to the negatively charged bladder mucosa. Thereby, NG/HCPT was given a mucosal adhesion. In addition, the amphiphilic nanogel with l-lysine residues will allow HCPT to enter cells similarly to amphiphilic cell–penetrating peptides (CPPs) (Shin et al., 2014). Cystine offers an opportunity that the disulfide bonds in the NG/HCPT core can be selectively degraded by intracellular GSH, which further accelerates the release of HCPT, thereby enhancing cell apoptosis (Wang J. et al., 2014; Lin TY. et al., 2016). This feature can reduce the dosage and avoid the potential risk of drug resistance due to high-dose administration. Based on this, Guo et al. (Guo et al., 2020) prepared a positively charged disulfide bond cross-linked nanogel, oligoarginine-PEG-P (LP-co-LC) (R9-PEG-P (LP-co-LC)), which further increases the residence time and enhanced the penetration ability of chemotherapeutics into the bladder wall. CPPs were less cytotoxic and can penetrate through the cell membrane to transport the “cargo” (Douat et al., 2015; Meloni et al., 2015). The combination of CPPs and antitumor drugs is becoming an attractive opinion to enhance the treatment effect (Shi and Qi, 2017). Arginine-rich CPPs have been confirmed with a high-efficient cellular uptake, especially the oligoarginine which contains nine arginine residues (Li et al., 2015; Zhang N. et al., 2016). Therefore, R9 upregulates the mucosal adhesion of the HCPT-loaded R9-PEG-P (LP-co-LC) (R9 NG/HCPT) through an electrostatic interaction with the negatively charged bladder, further promoting the permeability of the drug-loaded nanogel in the bladder wall. In addition, as a CPP, R9 effectively penetrates the cell membrane and transports the drugs. The disulfide bond gives the nanogel the function of selectively delivering HCPT in the cell through the reducing microenvironment. R9 NG/HCPT exhibits excellent cytotoxicity to human BC 5637 cells *in vitro* and significantly enhances the antitumor activity in mouse and rat orthotopic BC models *in vivo*.

Chen et al. (Chen et al., 2017b) synthesized morpholine and phenylboronic acid dual-modified polypeptide nanogels (PMNG) to conform receptor-mediated targeting and microenvironment-mediated targeting into a drug delivery system. Phenylboronic acid was used for selective receptor-mediated targeting in the highly metastatic cells, overexpressing sialyl on the membrane (Matsumoto et al., 2010; Sanjoh et al., 2014). Charge-transformable morpholine was neutral at physiological pH, and it has a positive charge in tumor tissues, which can be used to enhance cellular internalization in the tumor microenvironment (Zhang Y. et al., 2013; Guo et al., 2015). The nanogels were cross-linked by the core of disulfide bonds, which can be disassembled by intracellular GSH to selectively release DOX. PMNG has an excellent ability to target primary and metastatic B16F10 tumors, overexpressing sialyl *in vitro* and *in vivo*. In addition, PMNG/DOX has shown great advantages in inhibiting the growth of primary and metastatic tumors.

Xing et al. (Xing et al., 2012) prepared reduction-sensitive polymer nanocarriers with near-infrared fluorescent probes. They first synthesized a disulfide bond cross-linked polypeptide nanogel (NIRF nanogels) with near-infrared fluorescence properties and then encapsulated DOX in the core of the nanogels to prepare a near-infrared fluorescent drug carrier (NIRF prodrug). *In vitro* drug release studies of NIRF prodrugs have shown that there was an accelerated release in the environment where exists 10 mmol/L GSH. Studies on cell uptake of NIRF nanogels and NIRF prodrugs have indicated that they can enter cells through cellular endocytosis. NIRF labeling can directly describe the drug release from the NIRF nanogels using imaging methods, and subsequently the released drug molecules migrate to the nuclei, while the NIRF nanogel remains in the cytoplasm. The *in vivo* distribution of NIRF nanogels and NIRF prodrugs on tumor-bearing mice indicates that they all accumulate at the tumor site 24 h after injection through enhanced retention effect and permeability. This accumulation may be attributed to the highly permeable vascular structure of the tumor, which leads to the accumulation of nanomaterials (Ichikawa et al., 2004). The NIRF prodrugs that were prepared in these studies have antitumor potential.

The third strategy is to oxidize polypeptides containing sulfhydryl groups to prepare disulfide bond cross-linked polypeptide nanogels.

**FIGURE 7:**
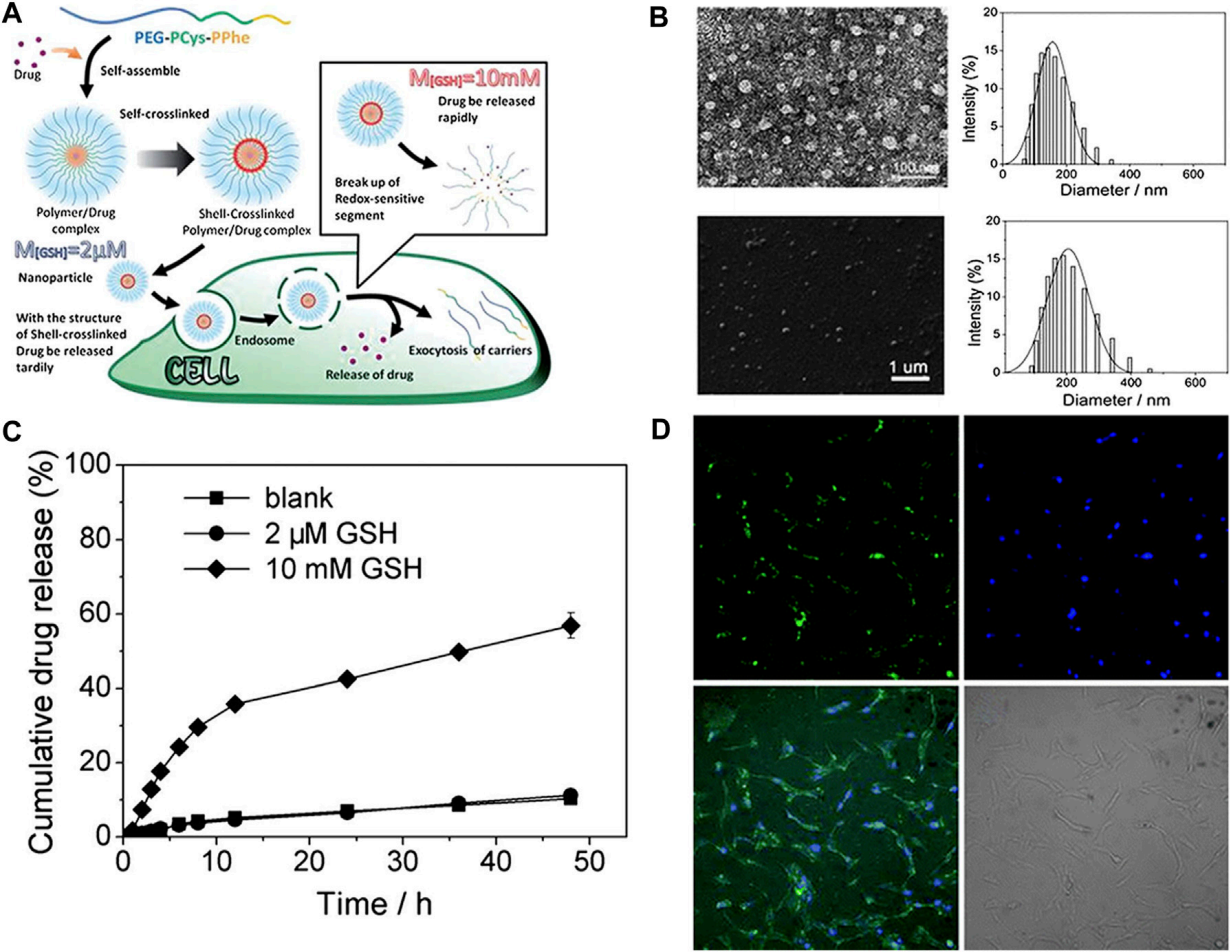
Characterization of self-assembly process and drug release of the nanogels (Wang et al., 2012). **(A)** Self-assembly process and drug release of the nanogels. **(B)** TEM image and particle size distribution of nanogels and the scanning electron microscope image and particle size of nanogels in the tetrahydrofuran solution. **(C)** Drug release of PEG-PCys-PPhe. **(D)** Confocal laser scanning microscopy (CLSM) image regarding co-incubation of HeLa cells and PEG-PCys-PPhe.

Wang et al. (Wang et al., 2012) prepared poly (ethylene glycol)-b-poly (l-cysteine)-b-poly (l-phenylalanine) (PEG-PCys-PPhe) by ROP. As shown in Figure 7A, self-assembled into a micelle with a core shell structure in an aqueous solution, the shell has self-crosslinked by the oxidation of the thiol group, and finally a disulfide-bonded cross-linked nanogel with reduction responsiveness was obtained. Glue, even in harsh environments can maintain the stability of the nanogels. As shown in Figure 7B, the hydrodynamic radius measured by DLS was about 150 nm, whereas the average diameter measured by TEM was about 50 nm. As shown in Figure 7C, *in vitro* drug release studies have shown that nanogels can reduce drug loss and accelerate drug release due to high GSH levels in the cell. As shown in Figure 7D, the cell uptake experiment results showed that the nanogels can be successfully internalized into HeLa cells because all cells exhibited green fluorescence.

In summary, because the disulfide bond contained in cystine can be quickly broken under a high GSH environment, thereby releasing a large amount of drug, this method can greatly improve the therapeutic efficiency of the drug.

#### Reactive Oxygen Species–Responsive Nanogels

ROS play an important role in cell signal transduction. They are involved in various signal paths, such as mediating inflammation, cell growth and differentiation, and regulating enzyme activity. Reactive oxygen species mainly exist in hydrogen peroxide, superoxide, and hydroxyl radicals (Lee et al., 2013) and are highly reactive. It is worth noting that normal cells have a stable redox environment and have a series of systems to remove and balance ROS levels. An increase in the ROS level may destroy intracellular homeostasis and cause oxidative damage to lipase and DNA, thereby leading to a series of diseases. Cancer cells with abnormal regulation of redox homeostasis and stress adaptability are more susceptible to oxidative stress induced by ROS generators, which is a significant basis for ROS therapy to work. ROS-responsive drugs can kill cancer cells selectively and reduce the damage to normal cells. At present, ROS-responsive nanodrugs, microspheres, and polymers have been well-studied. However, there are only a few reports on ROS-responsive nanogels (Liu JN. et al., 2017; Bawa and Oh, 2017; Dharmaraja, 2017).

**FIGURE 8:**
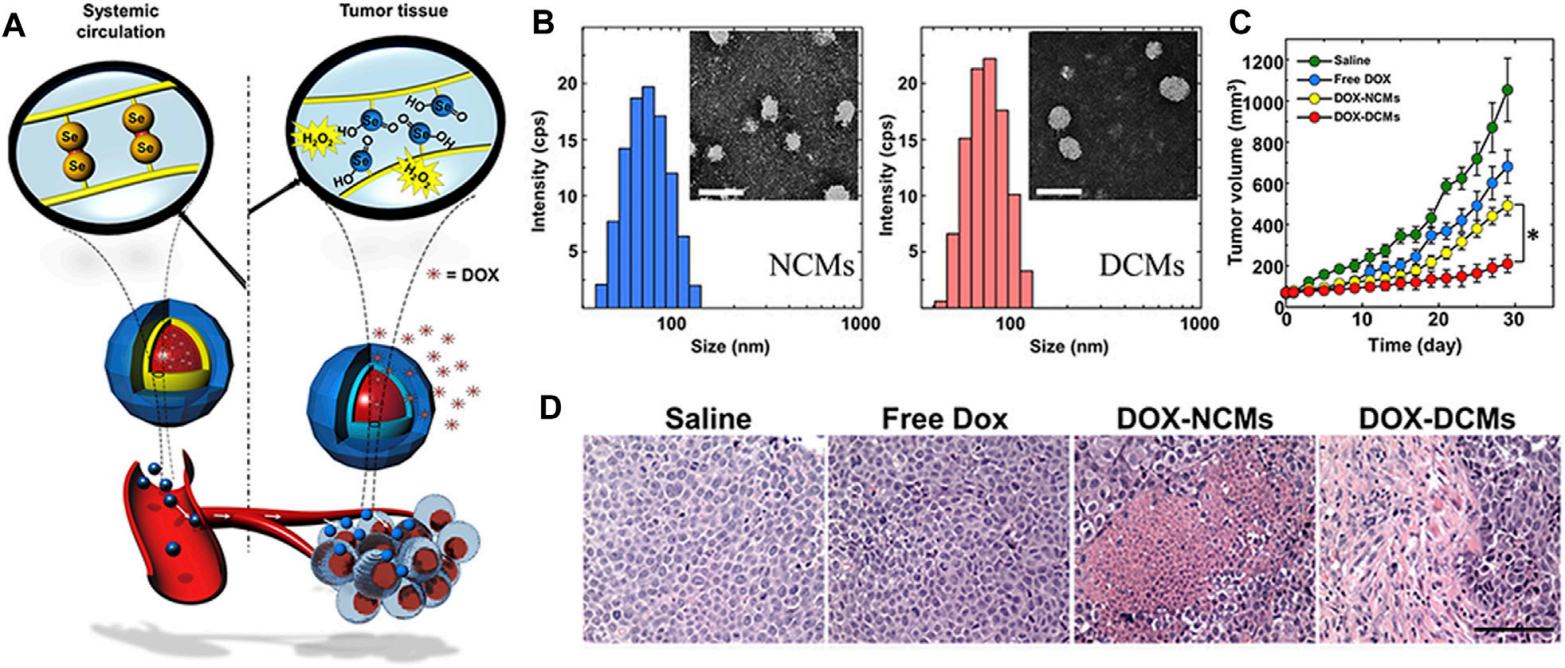
Characterization and treatment results of *in situ* DCMs (Deepagan et al., 2016). **(A)** DCMs maintained stability in circulation and the schematic diagram regarding DCMs triggering drug release by lysis in the tumor tissue. **(B)** Size distribution of NCM and DCMs. **(C)** Tumor growth curve. **(D)** H &E staining images of tumor tissue.

Deepagan et al. (Deepagan et al., 2016) developed *in situ* diselenide–cross-linked nanogels (DCMs) that, as anticancer drug carriers, can respond to abnormal ROS levels in tumor areas. The DCMs were derived spontaneously from selenol-loading triblock copolymers that were composed of polyethylene glycol and polypeptide derivatives. As shown in Figure 8A, DOX was effectively encapsulated in the hydrophobic core during the formation of nanogels. The DCMs maintained their stability in blood. However, when DCMs enter into ROS-rich tumor tissues, the hydrophobic diselenide bond of the DCMs was broken into hydrophilic selenic acid derivatives, which triggers the drug release from DOX-encapsulating DCMs (DOX-DCMs). As shown in Figure 8B, both DCMs and non-crosslinked micelles (NCMs) were spherical with the feature of a single peak size distribution. The average diameter of NCM and DCMs was about 65.48 and 85.10 nm, respectively. Least absorption of these nanoparticles by the reticuloendothelial system was observed (Blanco et al., 2015). The DCMs have great structural stability, even in the presence of destabilizing agents, and the DCMs maintained structural stability for at least 6 days under physiologic conditions. The diselenide cross-linking of DOX-DCMs, as a diffusion barrier, can effectively control the release of DOX. However, more DOX was released from DOX-DCMs within 3 days in the presence of H_2_O_2_ (100 μM). Moreover, enhanced drug release inhibited the efflux of p-glycoprotein (Wang et al., 2011). As shown in Figure 8C, compared with other treatments, DOX-DCM treatment greatly reduced tumor volume. As shown in Figure 8D, the hematoxylin–eosin staining images confirmed that DOX-DCM treatment resulted in the presence of a larger amount of dead cells in tumors, but the damage to the main organs can be ignored.

#### Enzyme-Responsive Nanogels

The enzyme is a kind of biological-related stimulant that can be overexpressed at the edge of tumor invasion and can also be used as a trigger to control the release of the drug. Currently, the main targets are matrix metalloproteinase and cathepsin B.

**FIGURE 9:**
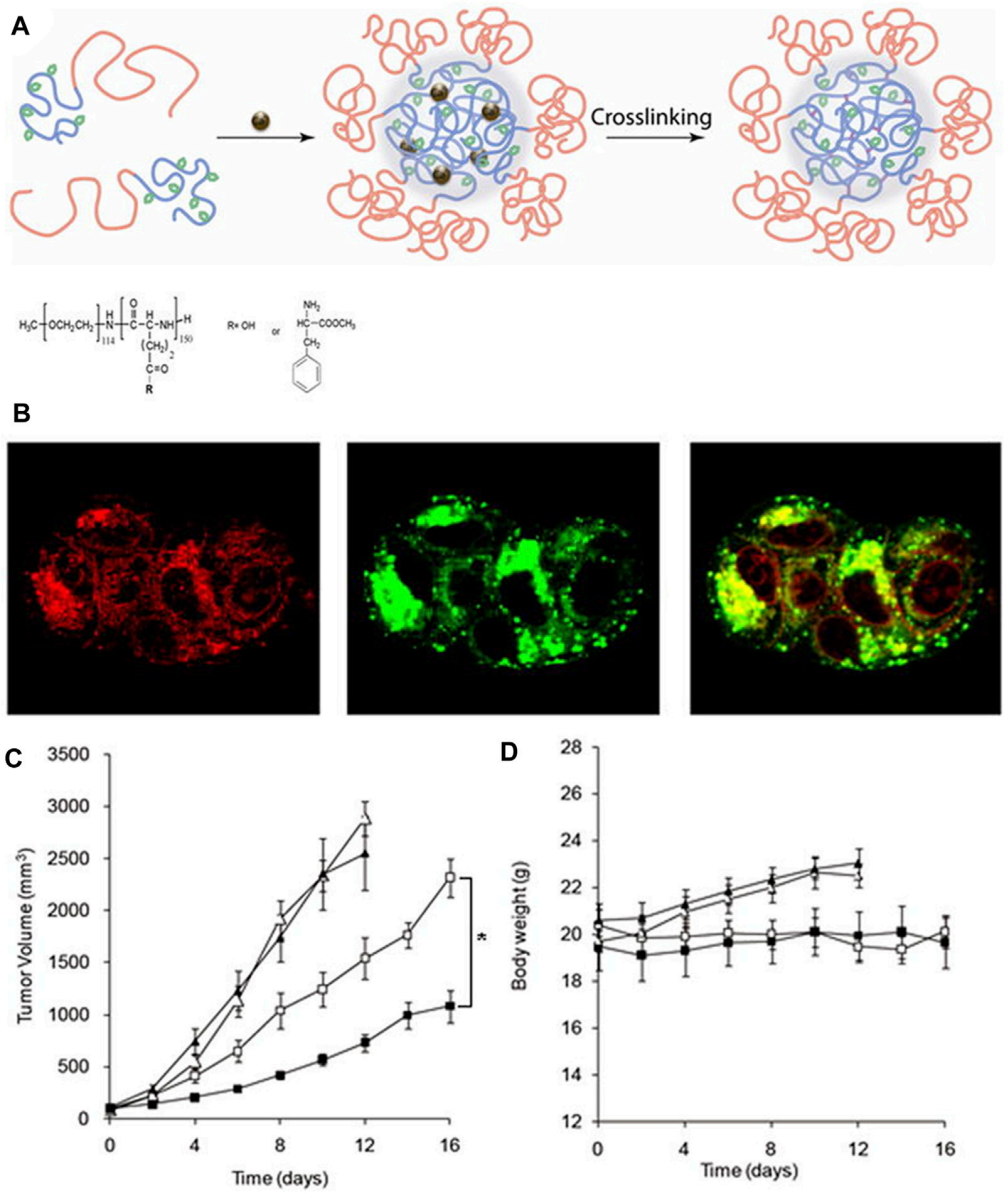
Synthesis and biological characterization of nanogels (Kim et al., 2013). **(A)** Schematic diagram of nanoparticle self-assembly. **(B)** Localization of DOX-loaded nanogels in cells. Effects of DOX-loaded nanogels on the tumor volume **(C)** and body weight **(D)**.

Kim et al. (Kim et al., 2013) synthesized partially hydrophobically modified polypeptide poly (ethylene glycol)-b-poly (l-glutamic acid) (PEG-b-PPGA) with l-phenylalanine methyl ester moieties and then used it for the synthesis of the template of nanogels. Then, Ca^2+^ was added to make it condense (Figure 9A). The resultant nanogels exhibited features that were similar to those of the hydrogels due to the protonation of the carboxyl group and the pH-dependent helical performance of the PPGA segment. Then, they loaded DOX onto the nanogel at high drug capacity. Under strong destabilization conditions (urea), nanogels maintained their firm structure, but they can be destroyed rapidly by enzymatic degradation. As shown in Figure 9B, the DOX-loaded nanogel can regulate the release of drugs and control the of the nanogel by lysosomal capture. DOX-loaded cross-linked nanogels show lower cytotoxic activity than free DOX. The reduction of cytotoxicity was consistent with the degradation of the nanogels. In addition, as shown in Figure 9C, biodegradable PEG nanogels provided sufficient DOX concentration to inhibit tumor growth in xenograft mouse models of ovarian cancer. Due to the retention effect and enhanced permeability, nanogel particles accumulated in solid tumors. The increase in the circulation time of nanogels (Oberoi et al., 2012) also increases the contact between the tumor and drug. Nanogels have stronger antitumor activity than free adriamycin. In addition, as shown in Figure 9D, there was no significant change between the body weight of the control and treatment groups, suggesting that all treatments were well tolerated.

Guo et al. (Guo et al., 2018) successfully synthesized a tri-block copolymer mPEG-Peptide-PCL, a kind of novel enzyme-responsive nanoparticle. The copolymer can respond to matrix metalloproteinase which is active and overexpressed in cancer, and, therefore, it can be used to guide targeted therapy. In this copolymer, the role of PCL was to load drugs. The peptide was used to target the tumor. The peptide GPLGIAGQ was designed to be degraded by matrix metalloproteinase-2 (Kratz et al., 2001). Cell-penetrating peptide R9 could promote the cellular uptake of nanoparticles (Wang HX. et al., 2014). PEGylation can improve the stability of the nanogel and extend its circulation time *in vivo*. *In vitro*, these scholars also evaluated the cumulative release rate of curcumin, as a model drug, under different pH conditions. In addition, they also assessed the cytotoxicity and cellular uptake in L929 and NSCLC A549 cells. *In vivo*, Guo et al. (Guo et al., 2018) characterized the selective targeting and biodistribution of mPEG-peptide-PCL using an *in vivo* imaging system because of the strong fluorescence effect of curcumin.

In summary, the enzyme-responsive nanogel is released in response to biological enzymes, which has very good biological targeting and safety properties.

### Exogenous Stimuli–Responsive Nanogels

Exogenous stimuli can also influence the structure of nanogels and promote the release of nanogels in a responsive manner. Compared with endogenous stimuli–responsive nanogels, exogenous stimuli–responsive nanogels can be better controlled, and, therefore, exogenous stimuli–responsive nanogels release drugs more accurately. Exogenous stimuli–responsive nanogels mainly consisted of temperature-responsive and photoresponsive nanogels. Similar to endogenous stimuli–responsive nanogels, the mechanism of exogenous stimuli–responsive nanogels is that the exogenous stimuli promote the drug release rapidly by disrupting the internal cross-linking network of polypeptide nanogels, which results in expansion or chemical bond breakage.

#### Temperature-responsive Nanogels

Nanogels can also be designed to release drugs under exogenous stimulation (especially temperature) (Gao and Dong, 2017). The molecular structure of temperature-responsive nanogels usually contains functional groups, such as amides, ether bonds, and hydroxyl groups. Nanogels can respond to changes in external temperature through rapid volume changes, and a discontinuous volume phase transition occurs at a specific temperature, leading to the controlled release of drugs from these nanogels (Lin J.-Y. et al., 2016). This temperature is called lower critical solution temperature (LCST). When the temperature is below the LCST, nanogels are in a swollen state. When the temperature is above the LCST, the nanogels rapidly lose water and shrunk, thereby exhibiting a response to temperature (Kujawa and Winnik, 2001).

Nguyen et al. (Nguyen et al., 2018) synthesized a thermosensitive copolymer heparin-Pluronic F127 to co-encapsulate cisplatin and curcumins to form a dual-drug delivery system. The interaction between hydrated cisplatin and curcumins and the properties of amphiphilic heparin-Pluronic F127 greatly improved not only the working efficacy of the controlled release system but also the drug loading efficiency. The anti-proliferative activity determined by *in vitro* and xenograft tumor tests revealed that in-depth studies were needed to investigate the uses of this delivery system against various cancers and drug-resistant cancers because of the synergistic activity of bioactive phytochemical and anticancer drugs in nanocarriers.

**FIGURE 10:**
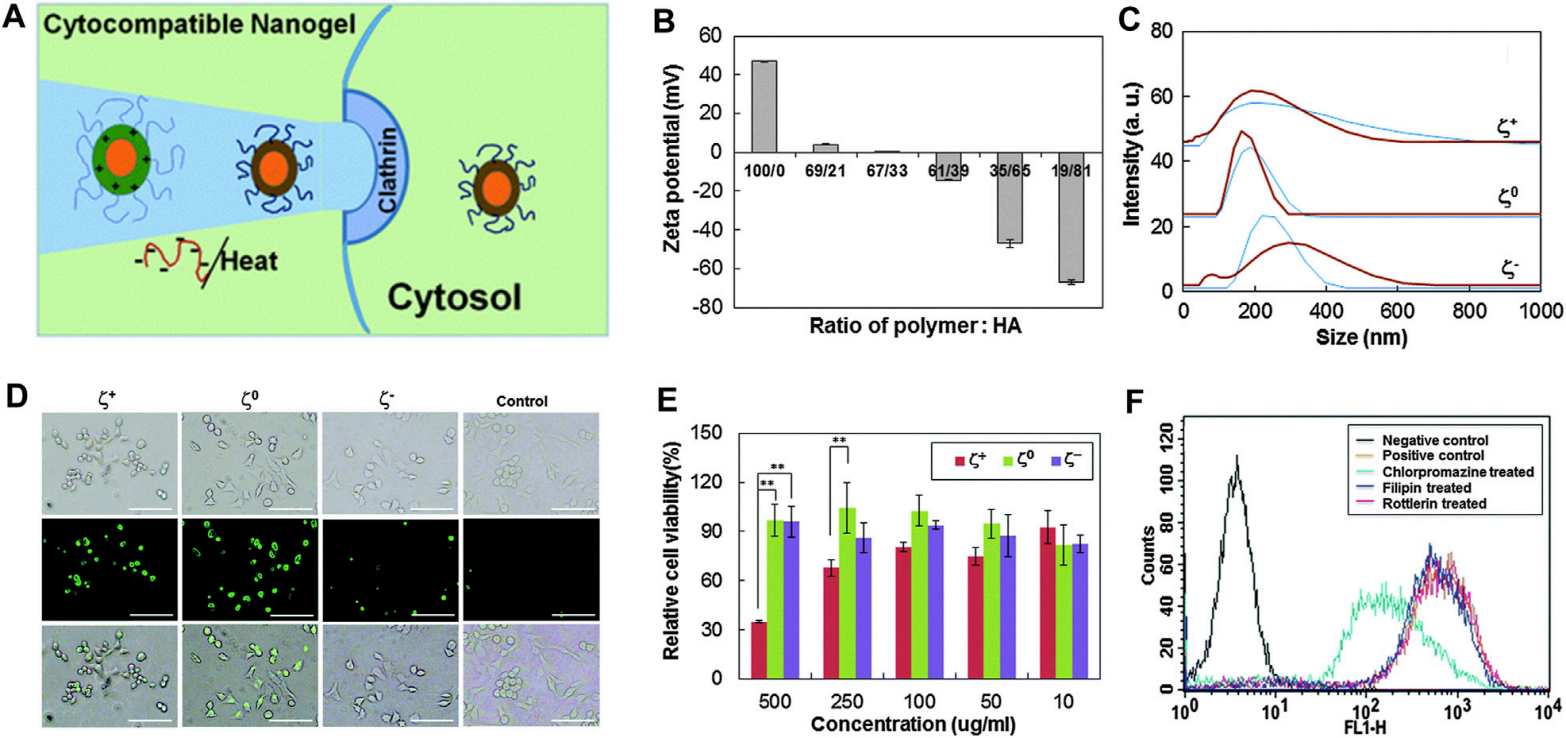
Physical and cellular characterization of temperature-sensitive nanocarriers (Ko et al., 2015). **(A)** Schematic diagram of temperature-sensitive nanocarriers. **(B)** ζ potential of nanogels prepared by PEG-PK-PA and HA). **(C)** Relationship between the particle size distribution of nanogels and different temperatures (blue curve for 20°Cand brown curve for 37°C). **(D)** Endocytosis after incubation for 12 h. **(E)** Relationship between the cytotoxicity of PEG-PK-PA/HA and the concentration of nanogels. **(F)** After inhibitor treatment, fluorescence-activated cell sorting data were compared to analyze the internalization of fluorescein isothiocyanate–conjugated bovine serum albumin–loaded nanogels with zero ζ potential (ζ^0^). Zero potential of ζ^+^, ζ^0^ , and ζ^−^ was +47 mV, 0, and −47 mV, respectively.

To deliver fluorescein isothiocyanate–conjugated bovine serum albumin, Ko et al.(Ko et al., 2015) designed temperature-sensitive tri-block nanogels using ionic complexes of hyaluronic acid (HA) and poly (ethylene glycol)-poly (l-lysine)-poly (l-alanine) which are called PEG-PK-PA. The PA block formed a hydrophobic nanocore. The positively charged PK block was compounded with the negatively charged HA block. PEG formed the outer shell of nanogels and prevented random aggregation of nanogels during the formation of ionic complexes. As shown in Figures 10A–C, nanogels have unique reversible temperature sensitivity. Therefore, nanogels shrink when heated. The internalization efficiency of nanogels could be controlled by adjusting the Zeta potential and size of nanogels and by greatly reducing the cytotoxicity of positively changed nanogels through the formation of ionic complexes with negatively charged HA. As shown in Figure 10D, the internalization of the model drug increased greatly if the ζ^+^ and ζ^0^ systems were used, and it was very low if the ζ^−^ system was used. Moreover, the use of the ζ^0^ system led to the most excellent internalization efficacy. As shown in Figure 10E, the MTT assay showed that the ζ^0^ and ζ^−^ systems have a significant improvement in cell compatibility compared with the ζ^+^ system. This occurred because the ionic interaction between the positively charged ζ^+^ system and the negatively charged cell membrane damages the cell membrane and induces cytotoxicity (Hunter, 2006; Kurosaki et al., 2009). Therefore, the negatively charged HA shield was combined with the positively charged PEG-PK-PA nanogel to reduce cytotoxicity. Filipin, rottlerin, and chlorpromazine are specific inhibitors of caveolae-mediated endocytosis, micropinocytosis, and clathrin-mediated endocytosis, respectively (Xiang et al., 2012; Saha et al., 2013). As shown in Figure 10F, flow cytometry results have confirmed that fluorescence was greatly reduced by the chlorpromazine-treated ζ^0^ system. This suggests that clathrin-mediated endocytosis led to the internalization of the ζ^0^ system.

Kim et al. (Kim et al., 2018) designed a biodegradable and thermoresponsive linear-dendritic nanogel containing poly (l-lysine), poly (l-lactic acid), and poly (N- isopropylacrylamide). The LCST of the nanogel was very low when it is in water at temperatures of 30–37°C. When the concentration is between 0.05 and 1 mg/ml (Kim et al., 2006), the integration of the poly (l-lactic acid) component provides the necessary hydrophobicity, and the drug reservoir was loaded with lipophilic reagents through hydrophobic–hydrophobic interactions. The nanogel has good biocompatibility. The nanogel itself and its degradative products were non-cytotoxic to neuron-like PC12 cells for at least 1 month. The nerve growth factors were added to the nanoparticles via aqueous phase mediation, which were slowly released from the nanogel for about 12 and 33 days at 25 and 37°C, respectively. The released nerve growth factor exhibits biological activity by promoting the neurite growth of PC12 cells. This study reveals a new concept that using biodegradable and thermoresponsive nanogels for the treatment of neurologic diseases through thermal targeting and sustained release of nerve growth factors and other protein drugs.

In summary, the temperature-sensitive nanogel is stimulated by external factors to achieve the purpose of the release and has better controllability.

#### Light-Responsive Nanogels

Light has become a stimulus that has attracted a lot of attention, is particularly suitable for the design of advanced biomedical platforms, improves the spatiotemporal control of the behavior of biomaterials, and dynamically adjusts their performance (Rapp and DeForest, 2020). In addition, the light-responsive platform also provides numerous opportunities for sequential degradation of implanted devices or remotely controlled drug delivery in a safe and non-invasive manner (Li et al., 2019).

**FIGURE 11:**
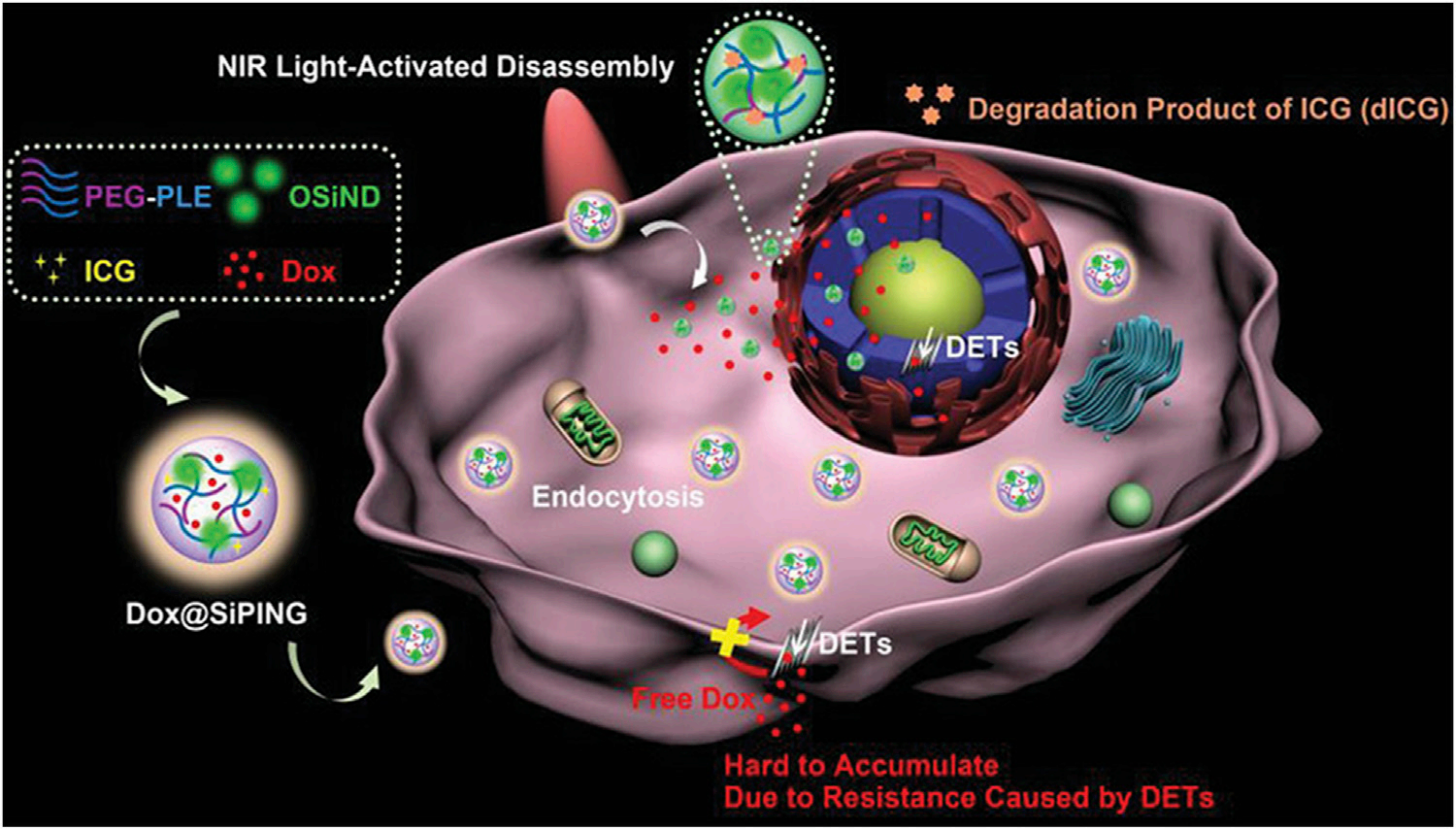
Schematic diagram of the nanogel structure. Such a structure can enhance cellular uptake and nuclear delivery of drugs, thereby contributing to the effective circumvention of multidrug resistance in cancer (Chen et al., 2019).

Inspired by the design of a “Trojan horse”, Chen et al. (Chen et al., 2019) developed a photocontrollable nanogel, which is called SiPING, through simple self-assembly of three functional materials. The SiPING was composed of three functional components, including 1) long-circulating biocompatible polymer poly (ethylene glycol)-b-poly (l-glutamic acid), which prevents the nanogels from being detected by drug efflux transporters (DETs) on the membrane to enhance the accumulation and circulation of the nanogels; 2) photodegradable indocyanine green, which enables the photoactivatable disassembly for photo-controllable drug release; and 3) green fluorescent organosilica nanodot, which can be used as the bridge connecting the other two components and bioimaging probes. The traceable “SiPING” plays the role of a “Trojan horse” and has a very broad application prospect as a multifunctional nanoplatform. As shown in Figure 11, due to the existence of DET, the plasma membrane acts as the gate of the cell, making it difficult for DOX to cross.

By hiding inside the nanogel with a suitable surface coating to avoid the recognition of DETs, lots of DOX molecules were transported into multidrug-resistant cells via endocytosis pathways. This allows DOX to form a high local concentration near the nucleus to prepare for further transport to the nucleus. Under the condition of photo-triggered degradation of the nanogels, a large amount of “auxiliaries” were released from the nanogels, allowing the drug to quickly occupy the nucleus under the condition of DETs and were still on present on the nuclear membrane; thus, multidrug resistance can be effectively circumvented. Therefore, the multidrug-resistant cells could be killed by DOX-loaded nanogels in two steps: 1) DOX-loaded nanogels’ behavior like a “Trojan horse” and escape from DETs on the plasma membrane, effectively transporting DOX into the cytoplasm and preventing drug efflux 2) Irradiation with near-infrared light leads to the decomposition of the nanogel, releasing a large number of DOX that can escape from DETs on the plasma membrane, thereby exerting its nuclear effect in multidrug-resistant cells.

There are few studies on light-responsive nanogels. Generally, photo responsiveness and thermal responsiveness are used in combination to achieve better results.

## Multi-Stimuli–Responsive Nanogels

To better use complex microenvironments in the tumor for drug delivery and release, more attention has been paid to multi-stimuli–responsive nanogels. The introduction of stimulus-responsive nanogels greatly improves the controllable degree and range of polymers, which can better deliver drugs to the tumor site and release drugs more accurately. Therefore, multiresponsive polymers have become the research area of most interest in recent years.

Zhang et al. (Zhang et al., 2019) prepared a polymer with disulfide bond–containing dimethyl l-cystinate and polycaprolactone oligomer using polycondensation via a pH-responsive imine bond. Then, they processed the polymer into nanoparticles (diameter <100 nm) using the nanoprecipitation method. The nanoparticles can release paclitaxel faster at mildly acidic pH and in the presence of high concentrations of GSH. They hardly exhibit release under physiological conditions. Compared with free paclitaxel, the nanoparticles were more cytotoxic to 4T1 cancer cells. *In vivo* test results show that the nanoparticles exhibit excellent anti-tumor ability and good biosafety.

Ding et al. (Ding et al., 2013) prepared reduction and pH dual-responsive polypeptide nanogels using the two-step strategy. First, using mPEG-NH_2_ as a macromolecule inducer, they synthesized mPEG-b-P (LGA-*co*-CELG) through the ROP of γ-benzyl-l-glutamate NCA (BLG-NCA) and γ-2-chloroethyl-l-glutamate NCA (CELG-NCA). Then, the benzyl group was deprotected (Ding et al., 2011b). Subsequently, the CELG unit was quaternized with 2,2′-dithiobis (N,N-dimethylethylamine) to prepare a polypeptide nanogel. DOX was added to the nanogels as an antitumor drug. Under normal physiologic conditions, DOX-loaded nanogels have a stable core cross-linking structure. However, in the simulated *in vivo* environment, tumor tissue, and intracellular redox microenvironment (10.0 nM GSH), carboxyl protonation reduces the interaction between the LGA unit in the core of nanoparticles and DOX and the breaking of the disulfide bond caused by GSH, which promotes the rapid release of effective load (Sanson et al., 2010). Confocal fluorescence microscopy results showed that compared with maternal DOX micelles and free DOX, DOX-loaded nanogels can deliver DOX to HepG2 cells more effectively. Quaternary ammonium group–induced improvement in cell internalization and low intracellular pH and high-level GSH triggered the enhancement of intracellular DOX release, ensuring the effective anti-proliferative activity of DOX-labeled nanogels *in vitro*. In addition, compared with DOX-loaded micelles and free DOX, DOX-loaded nanogels improved both *in vivo* and *in vivo* antitumor activity and *in vivo* safety.

Shi et al. (Shi et al., 2017) prepared a reduction and pH dual-responsive nanogel methoxy poly (ethylene glycol)-poly (l-glutamic acid-*co*-l-cystine). This nanogel has the advantage of simple preparation, high drug loading, and good response to different stimuli. These nanogels can be effectively synthesized by the ROP of amino acid NCA. The model antitumor drug DOX was added to the nanogels at the drug loading efficiency of 96.7 wt%. The particle size of NG/DOX was about 117.6 nm, which helps prolong the circulation time in the body and increases the accumulation in tumor tissues (Li et al., 2016). In addition, after intravenous injection, NG/DOX maintained structural integrity and minimal drug release in circulation *in vivo*. Due to the EPR effect, NG/DOX can be enriched in tumor tissues and enters the cell through endocytosis. NG/DOX releases effective load due to low intracellular pH and high-level GSH. Taken together, NG/DOX exhibits excellent antitumor properties and high biosafety *in vivo*. Some other antitumor drugs containing amino acids can be highly delivered as required by the smart nanogels. All these findings confirm that reduction and pH dual-responsive nanogels have bright application prospects to treat tumors.

Yi et al. (Yi et al., 2016) synthesized a smart peptide nanogel based on n-butylamine-poly (l-lysine)-b-poly (l-cystine) (PLL-PLC) with 1,2-dicarboxylic acid-cyclohexene anhydride (DCA) and folic acid (FA) for the delivery of multistage-responsive tumor-targeted drugs. The copolymer was spontaneously crosslinked with polymers to form pH and the redox nanoparticle FD-NP. The FD-NP has a reversible zeta potential of about 30 mV and +15 mV at pH 7.4 and 5.0, respectively. Moreover, the drug loading capacity of DOX in FD-NP was 15.7 wt%. It released approximately 24.5% DOX within 60 h at pH 7.4. However, at pH 5.0, the presence of 10 mM dithiothreitol significantly accelerated the release of DOX. FD-NP enhances the uptake of DOX in FA receptor–positive cancer cells only at pH7.4, but it promotes the uptake of DOX by FA receptor–positive HeLa and FA receptor–negative A549 cells in the weak acidic environment and greatly improves the cellular binding and lysosomal escapes. The *in vivo* study in a Hela cancer model revealed that compared with charge-irreversible FD-NPs, charge-reversible FD-NPs deliver DOX to the tumor more effectively. Therefore, the multistage-responsive FD-NPs can be used as highly efficient drug vectors for tumor-targeted chemotherapy.

Liu et al. (Liu L. et al., 2017) prepared self-crosslinked redox-responsive nanogels by coating DCA-grafted PEG-PLL (sPEG) with galactose-functionalized n-butylamine-poly (l-lysine)-b-poly (l-cysteine) polypeptides (GCL) and used them for TAM-targeted miR delivery and anticancer therapy. The *in vitro* study of Liu et al. (Liu L. et al., 2017) revealed that the cationic GLC core can be shielded by sPEG under physiologic pH conditions, but it was re-exposed because of rapid sPEG falling caused by reversible charge. The encapsulation of sPEG/GLC effectively promotes the delivery of macrophage-targeted miR under acidic conditions, but it reduced miR uptake at neutral pH. miR155-loaded sPEG/GLC (sPEG/GLC/155) nanocomplexes can increase miR155 expression in TAM both *in vivo* and *in vitro*. sPEG/GLC/155 can also increase the M1 macrophage markers and inhibit the M2 macrophage markers in TAM, effectively repolarizing the immunosuppressive TAM into antitumor M1 macrophages. In addition, sPEG/GLC/155 treatment greatly increases the number of activated T lymphocytes and natural killer cells in tumors, leading to tumor regression.

Wu et al. (Wu et al., 2016) successfully prepared plasmonic glyco-PEGylated polypeptide nanoparticles lactose (LAC) and PEG-grafted polycysteine terpolymer (PC-g-PEG-LAC) under mild conditions by integrating cocktail therapy and photothermal therapy into biodegradable and biocompatible nanocarriers. The polypeptide composite nanoparticles have strong near-infrared absorption and excellent photothermal properties. They have reductive responsiveness because their disulfide bonds can be degraded by GSH. They are also responsive to near-infrared light and can also release drugs. Irradiation heating can completely kill HepG2 cancer cells *in vitro*, showing excellent photothermal properties. The scholars loaded DOX and 6-mercaptopurine, two anticancer drugs, to nanoparticles through the Au–S bond and physical interaction, respectively. The composite nanoparticles loaded with both 6-mercaptopurine and DOX exhibit a reduction-sensitive and near-infrared light-triggered drug release profile and enhance cytotoxicity. The half-maximal inhibitory concentration produced by the cocktail chemo-photothermal therapy was lower than that of cocktail chemotherapy or photothermal therapy alone. Therefore, the cocktail chemo-photothermal therapy has a good synergistic antitumor effect. These findings provide evidence for establishing a simple strategy for the preparation of lactose-targeted, plasmonic, and dual drug–loaded polypeptide nanogels and open up a new way for developing a combined therapy of cocktail chemotherapy and photothermal therapy.

To realize spatiotemporal transmission and reverse hypoxia–induced drug resistance, Chen et al. (Chen et al., 2020) designed a shell-stacked nanoparticle (SNP) to co-encapsulate a proteasome inhibitor bortezomib (BTZ) and a vascular disrupting agent combretastatin A-4 phosphate (CA4P). The SNP is an ideal carrier for spatiotemporal transmission of BTZ and CA4P because it can rapidly detach the shell and penetrate tumor tissues (Chen et al., 2017a). First, BTZ was loaded onto the nanogel poly (l-lysine)-poly (l-phenylalanine-co-l-cystine) through hydrophobic interaction (NP_BTZ_), and then CA4P was electrostatically loaded on NP (_CA4P_NP_BTZ_). Finally, methoxy poly (ethylene glycol)-b-poly (l-lysine) modified with dimethylmaleic anhydride was coated on _CA4P_NP_BTZ_ to form a shell-stacked nanoparticle (S_CA4P_NP_BTZ_). After embedding CA4P and covering by the shell, the size of the composite increases to 150 nm due to the shell-stacking effect (Chen et al., 2017a). At high intracellular GSH concentrations, S_CA4P_NP_BTZ_ can spatiotemporally transport CA4P and BTZ to tumor blood vessels and tumor cells, respectively. Moreover, deeply penetrated NP can transport BTZ to tumor cells far away from the exudation site. In addition, the SNP can also reverse drug resistance caused by the overexpression of ABCG2 under the CA4P-induced hypoxia condition. Although the use of these nanoparticles can induce hypoxia in tumors, NP can reverse drug resistance by enhancing endocytosis and accelerating intracellular drug release. The spatiotemporal-targeted combination therapy significantly inhibits the progress of lung cancer and colon cancer. This provides a new strategy for the treatment of advanced cancer.

Multiresponse nanogels are currently a hot topic of research. Compared with single-response nanogels, it has better responsiveness and a better drug release effect.

## Prospects

To achieve more accurate and intelligent drug delivery to tumor tissues and improve multidrug resistance, scholars have made unremitting efforts in studying tumor microenvironment response. Although nanogels have advantages, including small particle size, long circulating time, good biocompatibility, biodegradability, and high drug loading capacity, there is still room for the optimization of nanoparticles in terms of single and multiple tumor microenvironment responses. Many study teams have performed *in vivo* and *in vitro* tests and confirmed that tumor microenvironment–responsive nanogels exhibit good therapeutic effects and have few adverse reactions. However, tumor microenvironment–responsive nanoparticles are easy to be eliminated by the immune system or accumulate toxicity. Therefore, they pose many challenges for the transition of tumor microenvironment–responsive nanoparticles from preclinical research to clinical application. With interdisciplinary development among materials science, molecular biology, tumor pharmacology, pharmaceutics, and other disciplines, future research studies should focus on nanogels with highly reversible, highly sensitive, and multiple responses.

## References

[B1] Abdullah-Al-Nahain N. Nam J. A. Mok H. Lee Y.-k. Park S. Y. (2013). Dual-responsive Crosslinked Pluronic Micelles as a Carrier to Deliver Anticancer Drug Taxol. Macromol. Res. 21 (1), 92–99. 10.1007/s13233-013-1011-z

[B2] Arroyo-Crespo J. J. Armiñán A. Charbonnier D. Balzano-Nogueira L. Huertas-López F. Martí C. (2018). Tumor Microenvironment-Targeted Poly-L-Glutamic Acid-Based Combination Conjugate for Enhanced Triple Negative Breast Cancer Treatment. Biomaterials 186, 8–21. 10.1016/j.biomaterials.2018.09.023 30278346

[B3] Bae Y. Kataoka K. (2009). Intelligent Polymeric Micelles from Functional Poly(ethylene Glycol)-Poly(amino Acid) Block Copolymers. Adv. Drug Deliv. Rev. 61 (10), 768–784. 10.1016/j.addr.2009.04.016 19422866

[B4] Bahadur K C R. Xu P. (2012). Multicompartment Intracellular Self-Expanding Nanogel for Targeted Delivery of Drug Cocktail. Adv. Mater. 24 (48), 6479–6483. 10.1002/adma.201202687 23001909

[B5] Bawa K. K. Oh J. K. (2017). Stimulus-Responsive Degradable Polylactide-Based Block Copolymer Nanoassemblies for Controlled/Enhanced Drug Delivery. Mol. Pharm. 14 (8), 2460–2474. 10.1021/acs.molpharmaceut.7b00284 28493712

[B6] Blanco E. Shen H. Ferrari M. (2015). Principles of Nanoparticle Design for Overcoming Biological Barriers to Drug Delivery. Nat. Biotechnol. 33 (9), 941–951. 10.1038/nbt.3330 26348965 PMC4978509

[B7] Chen J. Ding J. Wang Y. Cheng J. Ji S. Zhuang X. (2017a). Sequentially Responsive Shell-Stacked Nanoparticles for Deep Penetration into Solid Tumors. Adv. Mater. 29 (32), 1701170. 10.1002/adma.201701170 28632302

[B8] Chen J. Ding J. Xu W. Sun T. Xiao H. Zhuang X. (2017b). Receptor and Microenvironment Dual-Recognizable Nanogel for Targeted Chemotherapy of Highly Metastatic Malignancy. Nano Lett. 17 (7), 4526–4533. 10.1021/acs.nanolett.7b02129 28644032

[B9] Chen J. Jiang Z. Xu W. Sun T. Zhuang X. Ding J. (2020). Spatiotemporally Targeted Nanomedicine Overcomes Hypoxia-Induced Drug Resistance of Tumor Cells after Disrupting Neovasculature. Nano Lett. 20 (8), 6191–6198. 10.1021/acs.nanolett.0c02515 32697585

[B10] Chen J. Keltner L. Christophersen J. Zheng F. Krouse M. Singhal A. (2002). New Technology for Deep Light Distribution in Tissue for Phototherapy. Cancer J. 8 (2), 154–163. 10.1097/00130404-200203000-00009 11999949

[B11] Chen X. Zhang X. Guo Y. Zhu Y. X. Liu X. Chen Z. (2019). Smart Supramolecular "Trojan Horse"‐Inspired Nanogels for Realizing Light‐Triggered Nuclear Drug Influx in Drug‐Resistant Cancer Cells. Adv. Funct. Mater. 29 (13), 1807772. 10.1002/adfm.201807772

[B12] Deepagan V. G. Kwon S. You D. G. Nguyen V. Q. Um W. Ko H. (2016). *In Situ* diselenide-crosslinked Polymeric Micelles for ROS-Mediated Anticancer Drug Delivery. Biomaterials 103, 56–66. 10.1016/j.biomaterials.2016.06.044 27372421

[B13] Dharmaraja A. T. (2017). Role of Reactive Oxygen Species (ROS) in Therapeutics and Drug Resistance in Cancer and Bacteria. J. Med. Chem. 60 (8), 3221–3240. 10.1021/acs.jmedchem.6b01243 28135088

[B14] Ding J. Xiao C. Tang Z. Zhuang X. Chen X. (2011a). Highly Efficient "grafting from" an α-helical Polypeptide Backbone by Atom Transfer Radical Polymerization. Macromol Biosci. 11 (2), 192–198. 10.1002/mabi.201000238 20976724

[B15] Ding J. Xu W. Zhang Y. Sun D. Xiao C. Liu D. (2013). Self-reinforced Endocytoses of Smart Polypeptide Nanogels for "On-Demand" Drug Delivery. J. Control. Release 172 (2), 444–455. 10.1016/j.jconrel.2013.05.029 23742879

[B16] Ding J. Xiao C. Zhao L. Cheng Y. Ma L. Tang Z. (2011b). Poly(L -glutamic Acid) Grafted with Oligo(2-(2-(2-Methoxyethoxy)ethoxy)ethyl Methacrylate): Thermal Phase Transition, Secondary Structure, and Self-Assembly. J. Polym. Sci. A. Polym. Chem. 49 (12), 2665–2676. 10.1002/pola.24698

[B17] Dispinar T. Van Camp W. De Cock L. J. De Geest B. G. Du Prez F. E. (2012). Redox-responsive Degradable PEG Cryogels as Potential Cell Scaffolds in Tissue Engineering. Macromol Biosci. 12 (3), 383–394. 10.1002/mabi.201100396 22223302

[B18] Douat C. Aisenbrey C. Antunes S. Decossas M. Lambert O. Bechinger B. (2015). A Cell-Penetrating Foldamer with a Bioreducible Linkage for Intracellular Delivery of DNA. Angew. Chem. Int. Ed. Engl. 54 (38), 11133–11137. 10.1002/anie.201504884 26246005

[B19] Feng X. Xu W. Xu X. Li G. Ding J. Chen X. (2021). Cystine Proportion Regulates Fate of Polypeptide Nanogel as Nanocarrier for Chemotherapeutics. Sci. China Chem. 64 (2), 293–301. 10.1007/s11426-020-9884-6

[B20] Gao Y. Dong C.-M. (2017). Reduction- and Thermo-Sensitive Core-Cross-Linked Polypeptide Hybrid Micelles for Triggered and Intracellular Drug Release. Polym. Chem. 8 (7), 1223–1232. 10.1039/C6PY01929C

[B21] Gonçalves M. Maciel D. Capelo D. Xiao S. Sun W. Shi X. (2014). Dendrimer-Assisted Formation of Fluorescent Nanogels for Drug Delivery and Intracellular Imaging. Biomacromolecules 15 (2), 492–499. 10.1021/bm401400r 24432789

[B22] Guo F. Wu J. Wu W. Huang D. Yan Q. Yang Q. (2018). PEGylated Self-Assembled Enzyme-Responsive Nanoparticles for Effective Targeted Therapy against Lung Tumors. J. Nanobiotechnology 16 (1), 57. 10.1186/s12951-018-0384-8 30012166 PMC6048871

[B23] Guo H. Li F. Qiu H. Xu W. Li P. Hou Y. (2020). Synergistically Enhanced Mucoadhesive and Penetrable Polypeptide Nanogel for Efficient Drug Delivery to Orthotopic Bladder Cancer. Research (Wash D C) 2020, 8970135. 10.34133/2020/8970135 32832909 PMC7420878

[B24] Guo H. Xu W. Chen J. Yan L. Ding J. Hou Y. (2017). Positively Charged Polypeptide Nanogel Enhances Mucoadhesion and Penetrability of 10-hydroxycamptothecin in Orthotopic Bladder Carcinoma. J. Control. Release 259, 136–148. 10.1016/j.jconrel.2016.12.041 28062300

[B25] Guo X. Wei X. Jing Y. Zhou S. (2015). Size Changeable Nanocarriers with Nuclear Targeting for Effectively Overcoming Multidrug Resistance in Cancer Therapy. Adv. Mater. 27 (41), 6450–6456. 10.1002/adma.201502865 26401989

[B26] He H. Cattran A. W. Nguyen T. Nieminen A. L. Xu P. (2014). Triple-responsive Expansile Nanogel for Tumor and Mitochondria Targeted Photosensitizer Delivery. Biomaterials 35 (35), 9546–9553. 10.1016/j.biomaterials.2014.08.004 25154666 PMC4157076

[B27] He L. Li D. Wang Z. Xu W. Wang J. Guo H. (2016). L-Cystine-Crosslinked Polypeptide Nanogel as a Reduction-Responsive Excipient for Prostate Cancer Chemotherapy. Polymers (Basel) 8 (2). 10.3390/polym8020036 PMC6432546 30979130

[B28] Holmgaard R. B. Zamarin D. Li Y. Gasmi B. Munn D. H. Allison J. P. (2015). Tumor-Expressed Ido Recruits and Activates MDSCs in a Treg-dependent Manner. Cell Rep 13 (2), 412–424. 10.1016/j.celrep.2015.08.077 26411680 PMC5013825

[B29] Huang H. Jiang C. T. Shen S. Liu A. Gan Y. J. Tong Q. S. (2019). Nanoenabled Reversal of Ido1-Mediated Immunosuppression Synergizes with Immunogenic Chemotherapy for Improved Cancer Therapy. Nano Lett. 19 (8), 5356–5365. 10.1021/acs.nanolett.9b01807 31286779

[B30] Hunter A. C. (2006). Molecular Hurdles in Polyfectin Design and Mechanistic Background to Polycation Induced Cytotoxicity. Adv. Drug Deliv. Rev. 58 (14), 1523–1531. 10.1016/j.addr.2006.09.008 17079050

[B31] Huo M. Yuan J. Tao L. Wei Y. (2014). Redox-responsive Polymers for Drug Delivery: from Molecular Design to Applications. Polym. Chem. 5 (5), 1519–1528. 10.1039/c3py01192e

[B32] Ichikawa K. Hikita T. Maeda N. Takeuchi Y. Namba Y. Oku N. (2004). PEGylation of Liposome Decreases the Susceptibility of Liposomal Drug in Cancer Photodynamic Therapy. Biol. Pharm. Bull. 27 (3), 443–444. 10.1248/bpb.27.443 14993821

[B33] Kim J. O. Oberoi H. S. Desale S. Kabanov A. V. Bronich T. K. (2013). Polypeptide Nanogels with Hydrophobic Moieties in the Cross-Linked Ionic Cores: Synthesis, Characterization and Implications for Anticancer Drug Delivery. J. Drug Target. 21 (10), 981–993. 10.3109/1061186X.2013.831421 23998716 PMC4020517

[B34] Kim Y. S. Gulfam M. Lowe T. L. (2018). Thermoresponsive- Co-biodegradable Linear-Dendritic Nanoparticles for Sustained Release of Nerve Growth Factor to Promote Neurite Outgrowth. Mol. Pharm. 15 (4), 1467–1475. 10.1021/acs.molpharmaceut.7b01044 29320631

[B35] Kim Y. S. Gil E. S. Lowe T. L. (2006). Synthesis and Characterization of Thermoresponsive-Co-Biodegradable Linear−Dendritic Copolymers. Macromolecules 39 (23), 7805–7811. 10.1021/ma0602730

[B36] Ko D. Y. Moon H. J. Jeong B. (2015). Temperature-sensitive Polypeptide Nanogels for Intracellular Delivery of a Biomacromolecular Drug. J. Mater. Chem. B 3 (17), 3525–3530. 10.1039/C5TB00366K 32262236

[B37] Kratz F. Drevs J. Bing G. Stockmar C. Scheuermann K. Lazar P. (2001). Development and *In Vitro* Efficacy of Novel MMP2 and MMP9 Specific Doxorubicin Albumin Conjugates. Bioorg. Med. Chem. Lett. 11 (15), 2001–2006. 10.1016/S0960-894X(01)00354-7 11454467

[B38] Kujawa P. Winnik F. M. (2001). Volumetric Studies of Aqueous Polymer Solutions Using Pressure Perturbation Calorimetry: A New Look at the Temperature-Induced Phase Transition of poly(N-Isopropylacrylamide) in Water and D2O. Macromolecules 34 (12), 4130–4135. 10.1021/ma002082h

[B39] Kuppusamy P. Li H. Ilangovan G. Cardounel A. J. Zweier J. L. Yamada K. (2002). Noninvasive Imaging of Tumor Redox Status and its Modification by Tissue Glutathione Levels. Cancer Res. 62 (1), 307–312. 11782393

[B40] Kurosaki T. Kitahara T. Fumoto S. Nishida K. Nakamura J. Niidome T. (2009). Ternary Complexes of pDNA, Polyethylenimine, and Gamma-Polyglutamic Acid for Gene Delivery Systems. Biomaterials 30 (14), 2846–2853. 10.1016/j.biomaterials.2009.01.055 19232715

[B41] Lal S. Clare S. E. Halas N. J. (2008). Nanoshell-Enabled Photothermal Cancer Therapy: Impending Clinical Impact. Acc. Chem. Res. 41 (12), 1842–1851. 10.1021/ar800150g 19053240

[B42] Lee S. H. Gupta M. K. Bang J. B. Bae H. Sung H. J. (2013). Current Progress in Reactive Oxygen Species (ROS)-Responsive Materials for Biomedical Applications. Adv. Healthc. Mater. 2 (6), 908–915. 10.1002/adhm.201200423 25136729 PMC4146500

[B43] Li H. J. Du J. Z. Du X. J. Xu C. F. Sun C. Y. Wang H. X. (2016). Stimuli-responsive Clustered Nanoparticles for Improved Tumor Penetration and Therapeutic Efficacy. Proc. Natl. Acad. Sci. U S A. 113 (15), 4164–4169. 10.1073/pnas.1522080113 27035960 PMC4839420

[B44] Li L. Scheiger J. M. Levkin P. A. (2019). Design and Applications of Photoresponsive Hydrogels. Adv. Mater. 31 (26), e1807333. 10.1002/adma.201807333 30848524 PMC9285504

[B45] Li M. Schlesiger S. Knauer S. K. Schmuck C. (2015). A Tailor-Made Specific Anion-Binding Motif in the Side Chain Transforms a Tetrapeptide into an Efficient Vector for Gene Delivery. Angew. Chem. Int. Ed. Engl. 54 (10), 2941–2944. 10.1002/anie.201410429 25614369

[B46] Li M. Song W. Tang Z. Lv S. Lin L. Sun H. (2013). Nanoscaled poly(L-Glutamic Acid)/doxorubicin-Amphiphile Complex as pH-Responsive Drug Delivery System for Effective Treatment of Nonsmall Cell Lung Cancer. ACS Appl. Mater. Inter. 5 (5), 1781–1792. 10.1021/am303073u 23410916

[B47] Li S. Zhang T. Xu W. Ding J. Yin F. Xu J. (2018a). Sarcoma-Targeting Peptide-Decorated Polypeptide Nanogel Intracellularly Delivers Shikonin for Upregulated Osteosarcoma Necroptosis and Diminished Pulmonary Metastasis. Theranostics 8 (5), 1361–1375. 10.7150/thno.18299 29507626 PMC5835942

[B48] Li Y. Bui Q. N. Duy L. T. M. Yang H. Y. Lee D. S. (2018b). One-Step Preparation of pH-Responsive Polymeric Nanogels as Intelligent Drug Delivery Systems for Tumor Therapy. Biomacromolecules 19 (6), 2062–2070. 10.1021/acs.biomac.8b00195 29625005

[B49] Lin J.-Y. Lai P.-L. Lin Y.-K. Peng S. Lee L.-Y. Chen C.-N. (2016a). A Poloxamer-Polypeptide Thermosensitive Hydrogel as a Cell Scaffold and Sustained Release Depot. Polym. Chem. 7 (17), 2976–2985. 10.1039/C5PY02067K

[B50] Lin T. Y. Li Y. Liu Q. Chen J. L. Zhang H. Lac D. (2016b). Novel Theranostic Nanoporphyrins for Photodynamic Diagnosis and Trimodal Therapy for Bladder Cancer. Biomaterials 104, 339–351. 10.1016/j.biomaterials.2016.07.026 27479049 PMC5412594

[B51] Liu J. Pang Y. Huang W. Zhu Z. Zhu X. Zhou Y. (2011). Redox-responsive Polyphosphate Nanosized Assemblies: a Smart Drug Delivery Platform for Cancer Therapy. Biomacromolecules 12 (6), 2407–2415. 10.1021/bm2005164 21557536

[B52] Liu J. N. Bu W. Shi J. (2017a). Chemical Design and Synthesis of Functionalized Probes for Imaging and Treating Tumor Hypoxia. Chem. Rev. 117 (9), 6160–6224. 10.1021/acs.chemrev.6b00525 28426202

[B53] Liu L. Yi H. He H. Pan H. Cai L. Ma Y. (2017b). Tumor Associated Macrophage-Targeted microRNA Delivery with Dual-Responsive Polypeptide Nanovectors for Anti-cancer Therapy. Biomaterials 134, 166–179. 10.1016/j.biomaterials.2017.04.043 28463694

[B54] Liu X. Wang J. Xu W. Ding J. Shi B. Huang K. (2015). Glutathione-degradable Drug-Loaded Nanogel Effectively and Securely Suppresses Hepatoma in Mouse Model. Int. J. Nanomedicine 10, 6587–6602. 10.2147/IJN.S90000 26543363 PMC4622485

[B55] Lu C. Li B. Liu N. Wu G. Gao H. Ma J. (2014). A Hydrazone Crosslinked Zwitterionic Polypeptide Nanogel as a Platform for Controlled Drug Delivery. RSC Adv. 4 (92), 50301–50311. 10.1039/C4RA08871A

[B56] Ma N. Li Y. Xu H. Wang Z. Zhang X. (2010). Dual Redox Responsive Assemblies Formed from Diselenide Block Copolymers. J. Am. Chem. Soc. 132 (2), 442–443. 10.1021/ja908124g 20020681

[B57] Ma W. Chen Q. Xu W. Yu M. Yang Y. Zou B. (2021). Self-targeting Visualizable Hyaluronate Nanogel for Synchronized Intracellular Release of Doxorubicin and Cisplatin in Combating Multidrug-Resistant Breast Cancer. Nano Res. 14 (3), 846–857. 10.1007/s12274-020-3124-y

[B58] Mackiewicz M. Romanski J. Krug P. Mazur M. Stojek Z. Karbarz M. (2019). Tunable Environmental Sensitivity and Degradability of Nanogels Based on Derivatives of Cystine and Poly(ethylene Glycols) of Various Length for Biocompatible Drug Carrier. Eur. Polym. J. 118, 606–613. 10.1016/j.eurpolymj.2019.06.031

[B59] Matsumoto A. Cabral H. Sato N. Kataoka K. Miyahara Y. (2010). Assessment of Tumor Metastasis by the Direct Determination of Cell-Membrane Sialic Acid Expression. Angew. Chem. Int. Ed. Engl. 49 (32), 5494–5497. 10.1002/anie.201001220 20575125

[B60] Meloni B. P. Milani D. Edwards A. B. Anderton R. S. O'Hare Doig R. L. Fitzgerald M. (2015). Neuroprotective Peptides Fused to Arginine-Rich Cell Penetrating Peptides: Neuroprotective Mechanism Likely Mediated by Peptide Endocytic Properties. Pharmacol. Ther. 153, 36–54. 10.1016/j.pharmthera.2015.06.002 26048328

[B61] Meng F. Hennink W. E. Zhong Z. (2009). Reduction-sensitive Polymers and Bioconjugates for Biomedical Applications. Biomaterials 30 (12), 2180–2198. 10.1016/j.biomaterials.2009.01.026 19200596

[B62] Merino S. Martín C. Kostarelos K. Prato M. Vázquez E. (2015). Nanocomposite Hydrogels: 3D Polymer-Nanoparticle Synergies for On-Demand Drug Delivery. ACS Nano 9 (5), 4686–4697. 10.1021/acsnano.5b01433 25938172

[B63] Nguyen N. T. Nguyen N. N. T. Tran N. T. N. Le P. N. Nguyen T. B. T. Nguyen N. H. (2018). Synergic Activity against MCF-7 Breast Cancer Cell Growth of Nanocurcumin-Encapsulated and Cisplatin-Complexed Nanogels. Molecules 23 (12), 12. 10.3390/molecules23123347 PMC6321001 30567316

[B64] O'Connor T. P. Crystal R. G. (2006). Genetic Medicines: Treatment Strategies for Hereditary Disorders. Nat. Rev. Genet. 7 (4), 261–276. 10.1038/nrg1829 16543931

[B65] Oberoi H. S. Nukolova N. V. Laquer F. C. Poluektova L. Y. Huang J. Alnouti Y. (2012). Cisplatin-loaded Core Cross-Linked Micelles: Comparative Pharmacokinetics, Antitumor Activity, and Toxicity in Mice. Int. J. Nanomedicine 7, 2557–2571. 10.2147/IJN.S29145 22745537 PMC3383348

[B66] Park K. M. Lee D. W. Sarkar B. Jung H. Kim J. Ko Y. H. (2010). Reduction-sensitive, Robust Vesicles with a Non-covalently Modifiable Surface as a Multifunctional Drug-Delivery Platform. Small 6 (13), 1430–1441. 10.1002/smll.201000293 20564485

[B67] Peng J. Xiao Y. Li W. Yang Q. Tan L. Jia Y. (2018). Photosensitizer Micelles Together with Ido Inhibitor Enhance Cancer Photothermal Therapy and Immunotherapy. Adv. Sci. (Weinh) 5 (5), 1700891. 10.1002/advs.201700891 29876215 PMC5979747

[B68] Phillips D. J. Gibson M. I. (2014). Redox-Sensitive Materials for Drug Delivery: Targeting the Correct Intracellular Environment, Tuning Release Rates, and Appropriate Predictive Systems. Antioxid. Redox Signal. 21 (5), 786–803. 10.1089/ars.2013.5728 24219144

[B69] Ramasamy T. Haidar Z. S. Tran T. H. Choi J. Y. Jeong J. H. Shin B. S. (2014). Layer-by-layer Assembly of Liposomal Nanoparticles with PEGylated Polyelectrolytes Enhances Systemic Delivery of Multiple Anticancer Drugs. Acta Biomater. 10 (12), 5116–5127. 10.1016/j.actbio.2014.08.021 25169256

[B70] Rapp T. L. DeForest C. A. (2020). Visible Light-Responsive Dynamic Biomaterials: Going Deeper and Triggering More. Adv. Healthc. Mater. 9 (7), e1901553. 10.1002/adhm.201901553 32100475

[B71] Riddell S. R. (2001). Progress in Cancer Vaccines by Enhanced Self-Presentation. Proc. Natl. Acad. Sci. U S A. 98 (16), 8933–8935. 10.1073/pnas.171326398 11481463 PMC55350

[B72] Saha K. Kim S. T. Yan B. Miranda O. R. Alfonso F. S. Shlosman D. (2013). Surface Functionality of Nanoparticles Determines Cellular Uptake Mechanisms in Mammalian Cells. Small 9 (2), 300–305. 10.1002/smll.201201129 22972519 PMC4070423

[B73] Sanjoh M. Miyahara Y. Kataoka K. Matsumoto A. (2014). Phenylboronic Acids-Based Diagnostic and Therapeutic Applications. Anal. Sci. 30 (1), 111–117. 10.2116/analsci.30.111 24420252

[B74] Sanson C. Schatz C. Le Meins J. F. Soum A. Thévenot J. Garanger E. (2010). A Simple Method to Achieve High Doxorubicin Loading in Biodegradable Polymersomes. J. Control. Release 147 (3), 428–435. 10.1016/j.jconrel.2010.07.123 20692308

[B75] Satelli A. Li S. (2011). Vimentin in Cancer and its Potential as a Molecular Target for Cancer Therapy. Cell Mol Life Sci 68 (18), 3033–3046. 10.1007/s00018-011-0735-1 21637948 PMC3162105

[B76] Satelli A. Mitra A. Cutrera J. J. Devarie M. Xia X. Ingram D. R. (2014). Universal Marker and Detection Tool for Human Sarcoma Circulating Tumor Cells. Cancer Res. 74 (6), 1645–1650. 10.1158/0008-5472.Can-13-1739 24448245 PMC3959622

[B77] Shi B. Huang K. Ding J. Xu W. Yang Y. Liu H. (2017). Intracellularly Swollen Polypeptide Nanogel Assists Hepatoma Chemotherapy. Theranostics 7 (3), 703–716. 10.7150/thno.16794 28255361 PMC5327644

[B78] Shi N. Q. Qi X. R. (2017). Taming the Wildness of "Trojan-Horse" Peptides by Charge-Guided Masking and Protease-Triggered Demasking for the Controlled Delivery of Antitumor Agents. ACS Appl. Mater. Inter. 9 (12), 10519–10529. 10.1021/acsami.7b01056 28290666

[B79] Shin M. C. Zhang J. Min K. A. Lee K. Byun Y. David A. E. (2014). Cell-penetrating Peptides: Achievements and Challenges in Application for Cancer Treatment. J. Biomed. Mater. Res. A. 102 (2), 575–587. 10.1002/jbm.a.34859 23852939 PMC3929953

[B80] Stupp R. Mason W. P. van den Bent M. J. Weller M. Fisher B. Taphoorn M. J. (2005). Radiotherapy Plus Concomitant and Adjuvant Temozolomide for Glioblastoma. N. Engl. J. Med. 352 (10), 987–996. 10.1056/NEJMoa043330 15758009

[B81] Sun H. Meng F. Cheng R. Deng C. Zhong Z. (2013). Reduction-sensitive Degradable Micellar Nanoparticles as Smart and Intuitive Delivery Systems for Cancer Chemotherapy. Expert Opin. Drug Deliv. 10 (8), 1109–1122. 10.1517/17425247.2013.783009 23517599

[B82] Sundaramoorthy P. Ramasamy T. Mishra S. K. Jeong K. Y. Yong C. S. Kim J. O. (2016). Engineering of Caveolae-specific Self-Micellizing Anticancer Lipid Nanoparticles to Enhance the Chemotherapeutic Efficacy of Oxaliplatin in Colorectal Cancer Cells. Acta Biomater. 42, 220–231. 10.1016/j.actbio.2016.07.006 27395829

[B83] Tian H. Tang Z. Zhuang X. Chen X. Jing X. (2012). Biodegradable Synthetic Polymers: Preparation, Functionalization and Biomedical Application. Prog. Polym. Sci. 37 (2), 237–280. 10.1016/j.progpolymsci.2011.06.004

[B84] Wang F. Wang Y. C. Dou S. Xiong M. H. Sun T. M. Wang J. (2011). Doxorubicin-Tethered Responsive Gold Nanoparticles Facilitate Intracellular Drug Delivery for Overcoming Multidrug Resistance in Cancer Cells. ACS Nano 5 (5), 3679–3692. 10.1021/nn200007z 21462992

[B85] Wang H. X. Yang X. Z. Sun C. Y. Mao C. Q. Zhu Y. H. Wang J. (2014a). Matrix Metalloproteinase 2-responsive Micelle for siRNA Delivery. Biomaterials 35 (26), 7622–7634. 10.1016/j.biomaterials.2014.05.050 24929619

[B86] Wang J. Yang G. Guo X. Tang Z. Zhong Z. Zhou S. (2014b). Redox-responsive Polyanhydride Micelles for Cancer Therapy. Biomaterials 35 (9), 3080–3090. 10.1016/j.biomaterials.2013.12.025 24388799

[B87] Wang K. Luo G.-F. Liu Y. Li C. Cheng S.-X. Zhuo R.-X. (2012). Redox-sensitive Shell Cross-Linked PEG-Polypeptide Hybrid Micelles for Controlled Drug Release. Polym. Chem. 3 (4), 1084–1090. 10.1039/C2PY00600F

[B88] Wang Y. Xu S. Xiong W. Pei Y. Li B. Chen Y. (2016). Nanogels Fabricated from Bovine Serum Albumin and Chitosan via Self-Assembly for Delivery of Anticancer Drug. Colloids Surf. B Biointerfaces 146, 107–113. 10.1016/j.colsurfb.2016.05.043 27262260

[B89] Wei L. Chen J. Ding J. (2021). Sequentially Stimuli-Responsive Anticancer Nanomedicines. Nanomedicine (Lond) 16 (4), 261–264. 10.2217/nnm-2021-0019 33543644

[B90] Weigel P. H. Yik J. H. (2002). Glycans as Endocytosis Signals: the Cases of the Asialoglycoprotein and Hyaluronan/chondroitin Sulfate Receptors. Biochim. Biophys. Acta 1572 (2), 341–363. 10.1016/S0304-4165(02)00318-5 12223279

[B91] Wojtkowiak J. W. Verduzco D. Schramm K. J. Gillies R. J. (2011). Drug Resistance and Cellular Adaptation to Tumor Acidic pH Microenvironment. Mol. Pharm. 8 (6), 2032–2038. 10.1021/mp200292c 21981633 PMC3230683

[B92] Wu J. Liu X.-Q. Wang Y.-C. Wang J. (2009). Template-free Synthesis of Biodegradable Nanogels with Tunable Sizes as Potential Carriers for Drug Delivery. J. Mater. Chem. 19 (42), 7856–7863. 10.1039/B908768K

[B93] Wu X. Zhou L. Su Y. Dong C. M. (2016). Plasmonic, Targeted, and Dual Drugs-Loaded Polypeptide Composite Nanoparticles for Synergistic Cocktail Chemotherapy with Photothermal Therapy. Biomacromolecules 17 (7), 2489–2501. 10.1021/acs.biomac.6b00721 27310705

[B94] Xiang S. Tong H. Shi Q. Fernandes J. C. Jin T. Dai K. (2012). Uptake Mechanisms of Non-viral Gene Delivery. J. Control. Release 158 (3), 371–378. 10.1016/j.jconrel.2011.09.093 21982904

[B95] Xing T. Mao C. Lai B. Yan L. (2012). Synthesis of Disulfide-Cross-Linked Polypeptide Nanogel Conjugated with a Near-Infrared Fluorescence Probe for Direct Imaging of Reduction-Induced Drug Release. ACS Appl. Mater. Inter. 4 (10), 5662–5672. 10.1021/am301600u 22974285

[B96] Yan R. Liu X. Xiong J. Feng Q. Xu J. Wang H. (2020). pH-Responsive Hyperbranched Polypeptides Based on Schiff Bases as Drug Carriers for Reducing Toxicity of Chemotherapy. RSC Adv. 10 (23), 13889–13899. 10.1039/d0ra01241f PMC9051653 35492972

[B97] Yan S. Sun Y. Chen A. Liu L. Zhang K. Li G. (2017). Templated Fabrication of pH-Responsive Poly(l-Glutamic Acid) Based Nanogels via Surface-Grafting and Macromolecular Crosslinking. RSC Adv. 7 (24), 14888–14901. 10.1039/C7RA00631D

[B98] Yao J. He P. Zhang Y. Zhang H. Zhang P. Deng M. (2019). PEGylated Polylysine Derived Copolymers with Reduction‐responsive Side Chains for Anticancer Drug Delivery. Polym. Int. 68 (10), 1817–1825. 10.1002/pi.5892

[B99] Yi H. Liu P. Sheng N. Gong P. Ma Y. Cai L. (2016). *In Situ* crosslinked Smart Polypeptide Nanoparticles for Multistage Responsive Tumor-Targeted Drug Delivery. Nanoscale 8 (11), 5985–5995. 10.1039/c5nr07348k 26926103

[B100] Yu J. Fan H. Huang J. Chen J. (2011). Fabrication and Evaluation of Reduction-Sensitive Supramolecular Hydrogel Based on Cyclodextrin/polymer Inclusion for Injectable Drug-Carrier Application. Soft Matter 7 (16), 7386–7394. 10.1039/C1SM05426K

[B101] Zeng Q. Shao D. He X. Ren Z. Ji W. Shan C. (2016). Carbon Dots as a Trackable Drug Delivery Carrier for Localized Cancer Therapy *In Vivo* . J. Mater. Chem. B 4 (30), 5119–5126. 10.1039/C6TB01259K 32263509

[B102] Zhang L. Chen Y. Li Z. Li L. Saint-Cricq P. Li C. (2016a). Tailored Synthesis of Octopus-type Janus Nanoparticles for Synergistic Actively-Targeted and Chemo-Photothermal Therapy. Angew. Chem. Int. Ed. Engl. 55 (6), 2118–2121. 10.1002/anie.201510409 26732130

[B103] Zhang N. Yan Z. Zhao X. Chen Q. Ma M. (2016b). Efficient Mini-Transporter for Cytosolic Protein Delivery. ACS Appl. Mater. Inter. 8 (39), 25725–25732. 10.1021/acsami.6b08202 27632582

[B104] Zhang Q. Aleksanian S. Noh S. M. Oh J. K. (2013a). Thiol-responsive Block Copolymer Nanocarriers Exhibiting Tunable Release with Morphology Changes. Polym. Chem. 4 (2), 351–359. 10.1039/C2PY20582C

[B105] Zhang Q. Ding J. Lv C. Xu W. Sun X. Meng X. (2015). Epirubicin-Complexed Polypeptide Micelle Effectively and Safely Treats Hepatocellular Carcinoma. Polymers 7 (11), 2410–2430. 10.3390/polym7111521

[B106] Zhang X. Kang Y. Liu G. T. Li D. D. Zhang J. Y. Gu Z. P. (2019). Poly(cystine-PCL) Based pH/redox Dual-Responsive Nanocarriers for Enhanced Tumor Therapy. Biomater. Sci. 7 (5), 1962–1972. 10.1039/c9bm00009g 30810135

[B107] Zhang Y. Wang C. Xu C. Yang C. Zhang Z. Yan H. (2013b). Morpholino-decorated Long Circulating Polymeric Micelles with the Function of Surface Charge Transition Triggered by pH Changes. Chem. Commun. (Camb) 49 (66), 7286–7288. 10.1039/C3CC43334J 23846234

[B108] Zheng C. Li M. Ding J. (2021). Challenges and Opportunities of Nanomedicines in Clinical Translation. BIO Integration 2 (2), 57–60. 10.15212/bioi-2021-0016

[B109] Zheng P. Liu Y. Chen J. Xu W. Li G. Ding J. (2020). Targeted pH-Responsive Polyion Complex Micelle for Controlled Intracellular Drug Delivery. Chin. Chem. Lett. 31 (5), 1178–1182. 10.1016/j.cclet.2019.12.001

